# Main Factors Determining the Scale-Up Effectiveness of Mycoremediation for the Decontamination of Aliphatic Hydrocarbons in Soil

**DOI:** 10.3390/jof9121205

**Published:** 2023-12-16

**Authors:** Rafael Antón-Herrero, Ilaria Chicca, Carlos García-Delgado, Silvia Crognale, Davide Lelli, Romina Mariel Gargarello, Jofre Herrero, Anko Fischer, Laurent Thannberger, Enrique Eymar, Maurizio Petruccioli, Alessandro D’Annibale

**Affiliations:** 1 Department of Agricultural Chemistry and Food Science, Universidad Autónoma de Madrid, 28049 Madrid, Spain; rafael.anton@uam.es (R.A.-H.); enrique.eymar@uam.es (E.E.); 2 Novobiom, 1348 Ottignies-Louvain-la-Neuve, Belgium; ilaria@novobiom.com; 3 Department of Geology and Geochemistry, Universidad Autónoma de Madrid, 28049 Madrid, Spain; 4 Department for Innovation in Biological, Agri-Food and Forestry Systems, University of Tuscia, 01100 Tuscia, Italy; crognale@unitus.it (S.C.); davide.lelli@unitus.it (D.L.); petrucci@unitus.it (M.P.); dannib@unitus.it (A.D.); 5 Water, Air and Soil Unit, Eurecat, Centre Tecnològic de Catalunya, 08242 Manresa, Spain; rgargarello@gmail.com (R.M.G.); jofre.herrero@eurecat.org (J.H.); 6 Isodetect, 04103 Leipzig, Germany; fischer@isodetect.de; 7 VALGO, 76650 Petit-Couronne, France; laurent.thannberger@valgo.com

**Keywords:** mycoremediation, mycopile, aliphatic hydrocarbons, bioremediation, fungi, pollutants, microbiota, biopile

## Abstract

Soil contamination constitutes a significant threat to the health of soil ecosystems in terms of complexity, toxicity, and recalcitrance. Among all contaminants, aliphatic petroleum hydrocarbons (APH) are of particular concern due to their abundance and persistence in the environment and the need of remediation technologies to ensure their removal in an environmentally, socially, and economically sustainable way. Soil remediation technologies presently available on the market to tackle soil contamination by petroleum hydrocarbons (PH) include landfilling, physical treatments (e.g., thermal desorption), chemical treatments (e.g., oxidation), and conventional bioremediation. The first two solutions are costly and energy-intensive approaches. Conversely, bioremediation of on-site excavated soil arranged in biopiles is a more sustainable procedure. Biopiles are engineered heaps able to stimulate microbial activity and enhance biodegradation, thus ensuring the removal of organic pollutants. This soil remediation technology is currently the most environmentally friendly solution available on the market, as it is less energy-intensive and has no detrimental impact on biological soil functions. However, its major limitation is its low removal efficiency, especially for **l**ong**-c**hain **h**ydrocarbons (LCH), compared to thermal desorption. Nevertheless, the use of fungi for remediation of environmental contaminants retains the benefits of bioremediation treatments, including low economic, social, and environmental costs, while attaining removal efficiencies similar to thermal desorption. Mycoremediation is a widely studied technology at lab scale, but there are few experiences at pilot scale. Several factors may reduce the overall efficiency of on-site mycoremediation biopiles (mycopiles), and the efficiency detected in the bench scale. These factors include the bioavailability of hydrocarbons, the selection of fungal species and bulking agents and their application rate, the interaction between the inoculated fungi and the indigenous microbiota, soil properties and nutrients, and other environmental factors (e.g., humidity, oxygen, and temperature). The identification of these factors at an early stage of biotreatability experiments would allow the application of this on-site technology to be refined and fine-tuned. This review brings together all mycoremediation work applied to aliphatic petroleum hydrocarbons (APH) and identifies the key factors in making mycoremediation effective. It also includes technological advances that reduce the effect of these factors, such as the structure of mycopiles, the application of surfactants, and the control of environmental factors.

## 1. Introduction

Soil contamination by petroleum hydrocarbons (PH) is caused by spills, whether they are leaks, accidents, or loading and unloading operations, affecting soil structure and acidifying it [1]. The most efficient techniques for recovering soils contaminated with non-volatile hydrocarbons are soil washing and thermal treatments. The first method consists of excavating the soil and applying a wash using water with washing agents to remove, dissolve, or precipitate contaminants. Regarding the second method, thermal treatments involve the application of high temperatures, mostly from 90 °C to 600 °C, to volatilize organic contaminants or decompose them. Although these techniques decontaminate the soil, they are usually ex situ methods that damage the soil biologically and environmentally due to the extreme conditions it is subjected to. In addition, they are expensive and use much energy [2]. Landfill management, although currently used, should be discouraged as a decontamination strategy, as it involves the movement and sealing of contaminated soil, which results in a loss of resources (the soil itself) and does not remove the contamination [3].

Considering the growing scientific knowledge and recent guidelines for soil remediation and conservation that view soil as a finite non-renewable resource, more efficient and sustainable technologies need to be developed [4]. An alternative to physical and chemical treatments is offered by biological ones which exploit the power of microorganisms, autochthonous or allochthonous, to degrade and mobilize contaminants. Unlike the above-mentioned methods, biological treatments, or bioremediation as a general term, have several advantages, starting from their action on the nature of the soil and secondly due to their greener approach, in environmental and economic terms. On the other hand, it also presents some disadvantages. The first one is the relatively long time that the microorganisms often need to degrade the pollutants and recover the soil. There are two options to speed up the process: biostimulation and bioaugmentation. Biostimulation favors the conditions by amendments, nutrients, and/or bulking agents, to drive the multiplication and metabolism of the soil microbiota [1]. However, this can negatively affect pollutant removal because, as there is a higher concentration of these stimulants, fungi can use them as a preferred or alternative carbon source, leading to a decrease in the contaminant degradation [5].

The second strategy is known as bioaugmentation, where specialized microorganisms are added to biodegrade contaminants. Specialized microorganisms can be autochthonous or allochthonous, and they are chosen for their great capabilities to degrade certain pollutants. In terms of mycoremediation, the exploitation of fungi for bioremediation purposes is perfectly inserted. Thanks to their multicellular mycelium, fungi cover a large volume of soil and are resistant to high concentrations of toxins. They can extracellularly degrade a wide variety of organic contaminants through enzymes such as laccase, peroxidase, and manganese peroxidase [6]. Ligninolytic fungi degrade lignin with extracellular enzymes, producing water-soluble metabolites that are more bioavailable [7]. Also, it has been found that removal of polycyclic aromatic hydrocarbons (PAH) by non-ligninolytic enzymes was initiated by different PAH-specific and common upregulation of P450s, followed by downstream PAH-transforming enzymes such as epoxide hydrolases, dehydrogenases, FAD-dependent monooxygenases, dioxygenases, and glycosyl or glutathione transferases in white-rot fungus (WRF) [8].

For a successful mycoremediation process, fungi need to be in physical contact with the organic contaminant, which will depend on the properties of the soil, as well as the type of contaminant for bioavailability and bioaccessibility. In contrast to bacteria, fungi are actively able to move across unfavorable or toxic zones, to get in contact with substrates or nutrients. One of the great advantages of ligninolytic fungi is that they have extracellular enzyme systems that degrade non-bioavailable contaminants [9]. On the other hand, the synergisms between fungi and bacteria are important for maintaining great results of bioremediation at full scale. One of the most challenging aspects for scaling up is maintaining a stable fungal–bacterial community, which can be successfully established under non-axenic conditions and robust enough to simultaneously treat multiple contaminants [10].

Whereas mycoremediation seems to bring novel solutions for soil treatment, not much research has been conducted connecting the terms mycoremediation and aliphatic hydrocarbons, which are the most relevant contaminants for soils with spills of oil or petroleum-based products [11].

Figure S1 shows the prevailing occurrence of the terms “degradation and mycoremediation” over “aliphatic”, as denoted by the circle’s size and number of correlations. Notably, a bibliographic correlation emerges, associating mycoremediation with the implementation of *Agaricus bisporus* substrate for the comprehensive removal of total petroleum hydrocarbons (TPH). The pivotal keyword “bioaugmentation” is intricately linked to the augmentation of biodegradation processes. Furthermore, the keyword “biosurfactant” is within the same cluster as “crude oil” and “biodegradation.” The keyword “aliphatic hydrocarbons” finds placement in the red cluster, establishing connections with specific microbial genera and the term “diesel.” In aggregate, this figure underscores a discernible dearth of scholarly inquiry and associations within the literature, particularly concerning the mycoremediation of aliphatic hydrocarbons derived from petroleum.

The relatively low number of current patents for mycoremediation technologies (Table S1) illustrates the lack of knowledge transfer from basic research to higher Technology Readiness Levels (TRL), which is necessary for full-scale exploitation. Although the difficulty of patenting technologies based on bioprocesses is well known, the absence of registered methodologies at a large scale is evident. Even though patent number US2023014538 (A1) is the closest to an industrial application, it lacks explicit reference to aliphatic hydrocarbon treatment.

Given the lack of information about aliphatic hydrocarbon mycoremediation and the need to generate large scale applications, the main objective of this review will be to clarify the principal factors affecting the fungi and the scalability of the mycoremediation, as well as to elucidate advances in mycopile remediation of soil.

From now on, aliphatic petroleum hydrocarbons will be referred as “APH”, whereas “TPH” will only be used for all hydrocarbon fractions analyzed by quantitative methods that include all aliphatic and aromatic compound classes in range of *n*-alkanes from C_10_H_22_ to C_40_H_82_, isoalkanes, cycloalkanes, alkylbenzenes, alkylnaphthalenes, and polycyclic aromatic compounds. The abbreviation “PAH” will be used exclusively to refer to polycyclic aromatic hydrocarbons.

## 2. Factors That Affect the Success of the Mycoremediation

### 2.1. Bioavailability of Hydrocarbons

The bioavailability and bioaccessibility of contaminants are among the main factors governing the performance of soil bioremediation [12]. Bioavailability defines the fraction of contaminants that is freely available for microbial uptake and subsequent bioconversion at a given time. However, a particular contaminant might be made available over time or physically removed from the organism and, thus, bioaccessibility defines also what is potentially bioavailable [13,14,15].

A wide variety of intrinsic and extrinsic factors influence hydrocarbons’ bioavailability in soil. Intrinsic factors are the inherent chemical properties of aliphatic hydrocarbons, such as water solubility, lipophilicity, and the organic carbon/water partition coefficient (K_oc_). Conversely, extrinsic factors include the soil’s content and the form of the organic matter as well as the mineral fraction, physical interactions (e.g., sorption/desorption processes), environmental factors (temperature and rainfall), and hydrocarbon–soil contact time. APH interact with soil components through various sorption mechanisms, including covalent bonding and weak interactions, as well as diffusion such as into glassy or rubbery organic matter and pores of the mineral fraction [11,16]. The extent of these interactions largely depends on the inherent chemical structure of the pollutant, and both the amounts and properties of organic matter as well as the mineral fraction [11,16,17]. Sorption intensity increases as the contact time of the pollutant with soil constituents increases, a phenomenon termed “aging,” thus leading to a further reduction in pollutant bioavailability [18].

Due to the above-mentioned reasons, it is of paramount importance to quantify the fraction of contaminants which is available for microbial interactions and a wide array of chemical and biological methods have been developed for this purpose [19,20]. ISO 17402:2008 [21] was designed to establish a harmonized framework on the selection of methods for bioavailability assessment. It provides requirements and guidance to select methods to assess bioavailability for different target species regarding several classes of contaminants. Methods to assess bioavailability are not described. However, the main focus of this international standard relies on methods for the estimation of bioavailable concentrations of contaminants according to protection goals (i.e., risk assessment) and not on bioavailability assessment concerning the influence on inhibition of pollutant degradation by microorganisms (i.e., limitation of natural or stimulated biodegradation). Thus, ISO 17402:2008 [21] has limited application for evaluating bioavailability restrictions during microbial remediation, and methods should be considered that mimic conditions during bioremediation.

Chemical methods aim at determining the labile contaminants’ fraction (fast-desorbing fraction), assuming that only this pool is closely related to biodegradation [22]. Among chemical methods that have been tested for the determination of the bioavailability of aliphatic hydrocarbons (Table 1), a non-exhaustive extraction approach with milder solvents (e.g., *n*-propanol) than those conventionally used for extraction of hydrocarbons in the range of C_12_-C_40_ (e.g., dichloromethane; *n*-hexane-acetone) has been used successfully for predicting hydrocarbon biodegradation endpoints [23]. Another non-exhaustive extraction method, harnessing aqueous solutions of hydroxypropyl-β-cyclodextrin (HP-β-CD), gave reliable estimates of the bioaccessible fractions of C_10_-C_40_ hydrocarbons in aged, contaminated soils [23,24]. Another study, conducted with 11 historically contaminated soils, showed the capacity of the HP-β-CD method to yield reliable estimates of the biodegradable TPH fraction [25]. A further approach draws on a solid sorbent, namely Tenax, which, due to its very high sorption capacity and easy separability by floatation, has been successfully used in the determination of the labile fraction of a wide variety of persistent organic contaminants [22,26]. Ref. [25] found a high correlation between residual TPH concentrations after 24 h Tenax extraction and biodegradation in nine historically contaminated soils with values of the coefficients of determination (R^2^) higher than 0.92.

Also, sequential supercritical fluid extraction with CO_2_ has been used to determine the fast-desorbing APH fraction in a clay soil [29], and desorption data were found to fit strongly the two-site model proposed by [28].

Biological methods for measuring microbial bioavailability are based mostly on OECD 301 (A-F) standard tests [13], such as the determination of impact on biological oxygen demand variations in dissolved organic carbon removal (bioavailability) or assessing mineralization of ^14^C- or ^13^C-labelled target compounds (biodegradation) [30]. For the latter method, the amount of ^14^CO_2_ or ^13^CO_2_ produced from the catabolism of a ^14^C- or ^13^C-labeled target compound is taken as a measure of the bioavailability of the contaminant in soil [31,32,33]. Another approach for assessing pollutant bioavailability is based on bioreporters [34]. In most cases, genetically modified bacteria are used as whole-cell biosensors, which can respond with easily detectable signals when target compounds are present [12].

However, biodegradation rates may exceed abiotic desorption rates, suggesting that the process is not necessarily rate-limited by contaminant mobilization from the solid to the liquid phase [35]. Alkanes are often found as non-aqueous phase liquid around soil colloids, and direct contact of the microbial biomass with the contaminants cannot be ruled out [36]. This mechanism is certainly likely in filamentous fungi, for which the apical growth and the turgor pressure at the apex of the hyphae allow them to reach regions of the soil inaccessible to bacteria and to penetrate inside the aggregates [37]. For this purpose, fungi can ease the contact of bacteria and contaminants towards two different behaviors, defined as “fungal highways” and “fungal pipelines”. More details of these interactions will be discussed in Section 2.4.

In addition to their apical growth, filamentous fungi obtain an additional opportunity to come into close contact with the contaminants from the excretion of hydrophobins, low molecular weight and amphiphilic proteins [38]. These proteins tend to form amphipathic layers at the interfaces, thus affecting the wettability of the surfaces and mediating both adhesion and surface modification [39]. In this respect, the transcripts of fungal hydrophobin increased during the degradation of hydrocarbons in C bunker fuel oil by the white-rot fungus *Punctularia strigosozonata* [40]. This ability to modulate cell surface hydrophobicity impacts their ability to adhere to abiotic surfaces where hydrophobic contaminants accumulate [41,42]. Moreover, hydrophobins enable fungal hyphae to breach the air–liquid interface by reducing surface tension, thus allowing fungal access into unsaturated pores [43]. An additional role for these small amphipathic proteins is to favor biofilm settling via the stabilization of the adhesion of spores on hydrophobic surfaces and their ability to stabilize mature biofilms through their known ability to interact with glycans [44,45]. In developing biofilms, fungi offer anchoring sites to bacteria that allow them improved access to substrates with low bioaccessibility, as shown in several studies [46,47].

Another mechanism implemented by fungi to increase bioavailability of contaminants involves the release of pollutant-degrading enzymes into the extracellular environment. Interestingly, a group of extracellular heme-thiolate peroxidases, termed unspecific peroxygenases (E.C. 1.11.2.1, UPO), capable of modifying alkanes has been identified [48,49]. In particular, [49] found that a UPO from the white-rot fungus *Agrocybe aegerita* brought about the H_2_O_2_-dependent hydroxylation of saturated alkanes at the C_2_- and C_3_-position (C_3_-C_16_ range), giving rise to the formation of secondary alcohols; the same enzyme catalyzed the regioselective hydroxylation of cyclic alkanes. Another UPO from the white-rot fungus *Marasmius rotula* turned out to be able to initiate a cascade of mono- and di-terminal oxygenation reactions of several *n*-alkanes to carboxylic acids using H_2_O_2_ as the oxidant [48]. Fungi possess members of the cytochrome P450 monooxygenase (P450) superfamily [50], and some of them, especially CYP52 family members, are able to catalyze the terminal oxygenation of alkanes [51]. However, UPO, unlike P450, which are intracellular components, are secreted enzymes and, thus, alkane degradation is not rate-limited by alkane uptake and, accordingly, is made independent of bioavailability. The consultation of the Brenda enzyme database shows that, in addition to the two genera already mentioned, the production of UPO is also extended to other species relevant in the bioremediation of hydrocarbons, such as *Coprinopsis cinerea* and the ascomycete *Chaetomium globosum* [52].

Another mechanism implemented by fungi in the degradation of hydrocarbons involves the production of reactive oxygen species. This mechanism features the combined intervention of enzymatic reactions and chemical reactions, as shown by [53] in the degradation of trichloroethylene and perchloroethylene involving extracellular hydroxyl radicals (∙OH) produced via a quinone redox cycle. Although this mechanism has been described for the degradation of chlorinated aliphatics, it cannot be ruled out that it might operate on saturated alkanes, which are susceptible to hydroxyl radical-induced modifications [54].

In addition to these mechanisms, fungi can secrete amphiphilic substances which in turn are able to promote the mobilization of contaminants, termed surfactants. Several mechanisms can explain the surfactant-promoted increase in the bioavailability of persistent organic contaminants. The former, referred to as a roll-up mechanism, is due to the ability of the surfactant to promote a decrease in both surface and interface tensions, thus leading to a fostered transfer of the contaminant from the solid phase to the aqueous one [55,56]. Below the critical micelle concentrations (CMC), the surfactant monomers adsorbed on the soil’s colloid surface cause repulsion between the hydrophilic head group and the soil particles, thus leading to the desorption of hydrophobic contaminants from the soil particles. In addition, the wettability of the soil system changes because of the accumulation of surfactant monomers on soil/water and soil/contaminant interfaces. Once adsorption reaches saturation, namely under supra-CMC conditions, surfactant molecules start to aggregate, giving rise to the formation either of spheroidal or ellipsoidal micelles, and this phenomenon is associated with the solubilization of the contaminant within the hydrophobic cores of the micelles [57]. Some studies suggest a further mechanism whereby the biosurfactants help microorganisms adhere to the surface of soil particles housing the pollutant, thus reducing the path length of diffusion between the sorption and the biological uptake sites [58].

Surfactant-producing ability is widespread among fungi [59] and, in particular, among genera with hydrocarbonoclastic capacity [60]. Fungal biosurfactants have a wide structural variability which includes monoacyl glycerols, sophorose lipids, threalose lipids, methoxy phenyl oxime glycosides, and polymeric complexes (Table 2). Among them, the *Candida* species stand out for their ability to produce biosurfactants, some of which have been used for the cleanup of oil tanks, microbial enhanced oil recovery, and washing of oil-contaminated soil [61,62]. The surface-tension-reducing ability and the CMC values of some fungal surfactants valuably compete with those of bacterial origin, such as surfactin from *Bacillus subtilis* and rhamnolipids from *Pseudomonas aeruginosa* [58].

Of note is the fact that the use of synthetic surfactants in mycoremediation has mostly been applied to PAH-contaminated soil [63]. The lack of surfactant-assisted mycoremediation studies applied to oil-contaminated soils is somewhat surprising since the water solubilities of alkanes are comparatively lower than those of PAH; to exemplify, the solubility of hexadecane in water amounts to 9 × 10^−4^ mg/L, while that of five-ring PAH benzo[a]pyrene is four-fold higher (3.8 × 10^−3^ mg/L) [11]. This means that surfactant-promoted solubilization might be potentially beneficial to remediation performance even though some mobilizing agents can exert an adverse effect on growth of some fungi [63].

**Table 2:** Typology of fungal surfactants with respective surface tension and critical micelle concentration (CMC) values.

Organism	Phylum	Class	Typology of Surfactant	Surface Tension (mN/m)	CMC (mg/L)	Reference
*Candida (Starmerella) bombicola*	Ascomycota	Saccharomycetes	Sophorolipids	36.3–38.9 33.8	54–58 37.9	[64,65]
*Candida lipolytica*	Ascomycota	Saccharomycetes	Polymeric (lipo-protein polysaccharde complex)	32.0 30.0	1 × 10^4^ 2.5 × 10^4^	[61,66]
*Candida ishiwadae*	Ascomycota	Saccharomycetes	Monoacylglycerols	n.r.	n.r.	[67]
*Candida (Pseudozyma) anctartica*	Ascomycota	Saccharomycetes	Mannosylerythritol lipids	29.5	66.0	[68]
*Candida batistae*	Ascomycota	Saccharomycetes	Sophorolipids	39.3	138	[69]
*Candida bombicola*	Ascomycota	Saccharomycetes	Sophorolipids	37.0	108	[62]
*Candida spaherica*	Ascomycota	Saccharomycetes	Anionic glycolipids	25.0	250	[70]
*Torulopsis bombicola*	Ascomycota	Saccharomycetes	Sophorolipids	33.0	82	[71]
*Wickerhamomyces anomalus*	Ascomycota	Saccharomycetes	Glycolipid	29.2	0.9	[72]
*Aspergillus flavus*	Ascomycota	Eurotiomycetes	Methoxy phenyl oxime glycosides	20.0 25.0	170 80	[73]
*Aspergillus ustus*	Ascomycota	Eurotiomycetes	Polymeric (glyco-protein complex)	n.r.	n.r.	[74]
*Cladosporium resinae*	Ascomycota	Leotiomycetes	Glycolipid	35.0	n.r	[75]
*Fusarium fujikuroi*	Ascomycota	Sordariomycetes	Threalose lipid	27.0	30	[76]
*Penicillium chrysogenum*	Ascomycota	Eurotiomycetes	Polymeric (Lipopeptide)	n.r.	n.r.	[77]
*Cunninghamella echinulata*	Mucoromycota	Mucoromycetes	Polymeric (lipo-protein polysaccharde complex)	36	2.0 × 10^4^	[78]
*Mortierella alpina*	Mucoromycota	Mortierellomycetes	Hexapeptide	37.0	16	[79]
*Trichosporon asahii*	Basidiomycota	Tremellomycetes	Sophorolipids	30.0	197	[80]
*Ustilago maydis*	Basidiomycota	Ustilagomycetes	Mannosylerythritol lipids Cellobiose lipids	n.r.	n.r.	[81]
*Ceriporia (Irpex) lacerata*	Basidiomycota	Agaricomycetes	Mannosylerythritol lipids	31.1	n.r.	[82]
*Pleurotus djamor*	Basidiomycota	Agaricomycetes	Polymeric (lipo-protein polysaccharide complex)	28.8	1.0	[83]
*Pleurotus ostreatus*	Basidiomycota	Agaronmycetes	Polymeric (lipo-protein polysaccharide complex)	30.6	n.r.	[84]

### 2.2. Fungal Species and Metabolic Pathways

#### 2.2.1. Fungal Species Involved in Degradation of Aliphatic Hydrocarbons

Fungi are widespread in the soil matrix in pristine environments and in contaminated soils [9]. The functions they have, and their adaptation skills, change their distributions in the different environments. It is well known that fungi can degrade recalcitrant contaminants such as petroleum hydrocarbons, the aromatic/phenolic fractions in particular. Literature research in past decades was primarily focused on PAH degradation since they are more recalcitrant than aliphatic ones.

From an evolutionary point of view, the ability to degrade hydrocarbons by fungi may have developed in the context of biotrophic interactions in ecological niches characterized by a significant presence of abiotic and biotic sources of these compounds [85]. Starting from the “abiotic” sources, certain wood-inhabiting fungi developed the capability to degrade hydrocarbons due to the need to degrade lignin to get access to cellulose, which they use as a carbon source. These kinds of fungi are called ligninolytic fungi, or white-rot fungi, mostly belonging to the Basidiomycota phylum. These fungi utilize a non-specific battery of enzymes that recognize several contaminants, especially PAH and phenolic compounds, which exhibit lignin-like chemical structures. While it is quite common to find these kinds of fungi in pristine environments, it is less common to find them in contaminated sites where the presence of lignocellulosic materials is not necessarily ensured. Indeed, it is reported that in polluted soil, the most common fungal species (64%) belong to the phylum Ascomycota [9]. APH are undoubtedly easier to degrade than the aromatic and asphaltenes fractions, but they present some challenges as well. In fact, higher-molecular-weight, branched, and cyclic aliphatic hydrocarbons are water-repellent and are degraded more slowly than linear aliphatic hydrocarbons.

On the other hand, and concerning biotic sources, several organisms are capable of synthesizing alkanes and alkenes. Indeed, insects [86], higher plants [87], and cyanobacteria [88] are able to synthesize these compounds to protect themselves from environmental threats. It is no coincidence that entomophagous fungal species, such as *Metarrhizium anisopliae* and *Beauveria bassiana*, exhibit high hydrocarbonoclastic capacity even against long-chain alkanes due to the relevant presence of these compounds in the epicuticle of insects [89].

The ability of some fungi to grow on mixtures of long-chain alkanes (C_20_-C_40_) was already recognized in the first half of the 20th century and described by Zobell [90] in a comprehensive review, where *Aspergillus* and *Penicillium* were the most frequently mentioned genera. An upsurge of interest in APH fungal degraders took place in the second half of the 20th century as a consequence of the occurrence of accidents involving kerosene-fueled aircrafts due to the obstruction of the fuel supply circuits induced by the accumulation of fungal biomass [91,92]; among the fuel-contaminating fungal species, *Amorphotheca resinae*, currently named *Cladosporium resinae*, was most frequently detected. These fungal mats were able to exploit kerosene as a sole carbon source [93,94]. Other fungal genera were found in fuel tanks, including *Acremonium*, *Alternaria*, *Aspergillus*, *Aureobasidium*, *Botrytis*, *Candida*, *Cephalosporium (Acremonium)*, *Chaetomium*, *Chrysosporium*, *Cladosporium*, *Curvularia*, *Drechslera*, *Epicoccum*, *Geomyces*, *Geotrichum*, *Gliomastix*, *Fusarium*, *Hansenula*, *Helminthosporium* (most probably = *Drechslera*), *Humicola*, *Mucor*, *Paecilomyces*, *Penicillium*, *Pestalotiopsis*, *Phialophora*, *Phoma*, *Phomopsis*, *Pseudallescheria* (=*Scedosporium*), *Rhinocladiella*, *Rhizopus*, *Rhodotorula*, *Saccharomyces*, *Sordaria*, *Stemphylium*, *Thielavia*, *Trichoderma*, *Trichosporon*, *Trichothecium*, *Tritirachium*, and *Ulocladium* [91,95].

As mentioned above, the capacity to degrade petroleum hydrocarbons lies in the ecological niche and the mechanisms harbored by fungi due to this concern. In this frame, it is possible to divide the further explanations mainly into two parts, concerning the Ascomycota and Basidiomycota divisions, which also reflect the ecological niches and roles, and, consequently, the mechanisms, for hydrocarbon degradation. In natural and contaminated environments, Ascomycota species neatly predominate over those belonging to the phylum Basidiomycota and, to a greater extent, the Mucoromycota [36,96,97,98,99,100].

In contaminated sites, the presence of fungal specimens was largely reported as: *Fusarium*, *Penicillum*, *Aspergillus*, *Cephalosporium*, *Rhizopus*, *Paecilomyces*, *Torulopsis*, *Alternaria*, *Talaromyces*, *Gliocladium*, *Cladosporium*, *Geotrichum* belonging to the Ascomycota division, *Rhodotorula*, *Pleurotus* belonging to Basidiomycota, and *Mucor* belonging to Mucoromycota [101,102,103].

For example, during the isolation process, ref. [98] found a percentage of 56% of fungal isolates and 44% of bacterial ones from eight locations in Kazakhstan, and, among these fungal isolates, 78% belonged to Ascomycota and 22% to Basidiomycota. The isolation substrates were linear alkanes (tetradecane), branched alkanes (pristane), cyclic ketones (cyclohexanone), alkylphenol (4-tert-Amylphenol) and biphenyl. Tetradecane offered the largest number of isolates for both bacteria and fungi. Among the isolates, several species of *Fusarium* were retrieved, together with *Purpureocillium lilacinus*, *Sarocladium* sp., *Yarrowia lipolytica*, and *Candida parapsilosis*, belonging to Ascomycota; and *Rhodotorula mucilaginosa* and *Pseudozyma aphidis*, belonging to Basidiomycota. Fungal strains mostly grew on tetradecane and on the cyclohexanone (just *Fusarium*, *Candida*, and *Rhodotorula*).

Starting from the phylum of Ascomycota, the order Eurotiales encompasses the *Aspergillus* and *Penicillium* genera, several species of which are dominant members in microbial communities of crude-oil-impacted soils and sediments [96,100,104] and have a proven assimilatory capacity towards linear and branched aliphatic hydrocarbons [51,105,106,107]. Moreover, Eurotialean fungi display a high tolerance to hydrocarbon-induced stress as well as to other stressors; thus, they can be considered good candidates for the bioremediation of highly polluted sites, as shown in the next examples.

Husaini [108] isolated eighteen fungal strains identified as *Colletotrichum* (1), *Pestalotiopsis* (1), *Fusarium* (1) (both from the Amphisphaeriales order), *Trichoderma* (3, from the Hypocreales order), *Penicillium* (9), and *Aspergillus* (3) from the first 5 cm layer of soil, water, or a used motor oil sample from Kota Samarahan and Pending in Sarawak, Malaysia. The best candidates in terms of growth efficiency on motor oil plates were *Trichoderma* sp. (3 species, SA4-6), *Penicillium* (P1), and *Aspergillus* (P9).

Khan [109] studied three isolates (*Penicillium decumbens* PDX7, P. *janthinellum* SDX7, and *Aspergillus terreus* PKX4) from a petroleum-contaminated soil site in Anand, Gujarat, India and screened them for degradation of kerosene and diesel used as the sole carbon sources. By the end of the 16th day, they reached 97% by *Penicillium decumbens* PDX7, 94% by *P. janthinellum* SDX7, and 84% by *Aspergillus terreus* PKX4 for aliphatic fraction degradation in kerosene, and 80% by *Penicillium decumbens* PDX7, 76% by *P. janthinellum* SDX7, and 71% by *Aspergillus terreus* PKX4 for aliphatic fraction degradation in diesel oil. For kerosene degradation, it was observed that *n*-alkane fractions were easily degraded but the rate was lower for branched alkanes, followed by *n*-alkyl aromatics, cyclic alkanes, and PAH. For both kerosene and diesel oil, fungal degradation concerned mostly the aliphatic fraction. The researchers also found a high correlation between the production of enzymes such as laccase, manganese peroxidase, and dehydrogenase, and depletion of hydrocarbons (R = 0.78–0.82), indicating the involvement of these enzymes in the kerosene and diesel oil degradation.

Al-Hawash [110] reported that the two *Penicillium* isolates, RMA1 and RMA2, from the Rumaila oil field in Iraq, were able to perform alkane removal to different extents after 14 days. In fact, for RM1, the degradation of the *n*-alkane C_11_ was 100%, while 89% was achieved for RM2. The degradation extents decreased as the chain length increased, reaching up to 35% and 35% for RM1 and RM2, respectively, towards *n*-alkane C_25_ after 14 days’ incubation. These strains reduced surface tension when cultured on crude oil (1% *v*/*v*) and exhibited a cell surface hydrophobicity of more than 70%.

The ability to degrade higher alkanes appears to be a common feature also in the Hypocreales order, which, in addition to the *Acremonium*, *Fusarium*, *Trichoderma*, and *Thricotecium* genera, also includes either entomopathogenic species, such as *Metarhizium anisopliae* and *Beauveria bassiana*, or *Nematophagous* species, such as *Purpureocillium lilacinum*. However, despite their striking capacity to degrade long-chain hydrocarbons, entomopathogenic fungi are rather susceptible to environmental stresses [51,111], and, for this reason, their practical use is of limited relevance in bioremediation. Similarly, the ability of *P. liliacinum* to degrade alkanes and isoalkanes was verified on mineral liquid media [105,107] but has not so far been confirmed in contaminated soils. By contrast, species belonging to *Fusarium* and *Trichoderma* genera are often found in crude-oil-contaminated soils [96,100,112], and the indigenous isolates are often used to perform soil bioaugmentation [112].

Yanto [113] isolated 72 strains from rotted wood in Ehime Prefecture, Japan. These fungi were grown in asphalt-containing malt extract plates and selected for their capability to tolerate high concentrations of asphalt (1000 and 15,000 mg/L) added as a 1 mL or 2 mL solution with dichloromethane (DCM). The number of strains was drastically narrowed down to five isolates, among which the best was the Ascomycota *Pestalotiopsis* sp. NG007. *Pestalotiopsis* has been reported as a litter-decomposing ligninolytic fungus that is able to degrade lignin, tannins, melanins, humic substances, and cutin [114,115] and resins (50–60%) in 6 weeks [116]. The genus *Pestalotiopsis* has also been found to be able to completely degrade *n*-alkanes and branched C_15_-C_23_ alkanes in two months [108]. The order Saccharomycetales includes several hydrocarbonoclastic members belonging to the genera *Candida*, *Debaryomyces*, *Geotrichum*, *Hansenula (=Pichia)*, *Saccharomyces*, and *Yarrowia*. These members share properties such as an outstanding capacity to produce biosurfactants [62,72] and the presence of several CYP52 enzymes belonging to the cytochrome-P450 monooxygenase superfamily, which determine their ability to degrade alkanes [117,118].

A recent study conducted on the evolutionary history of a set of orthologous and paralogous CYP52 proteins from Saccharomycetales yeasts suggested the occurrence of frequent ancient and modern duplication and loss events yielding orthologous and paralogous groups. Docking analysis of deduced ancestral proteins within the CYP52 family suggested that the oxidation ability towards alkanes with chain length higher than C_10_ was a derived character, while the ancient function was the oxidation of lighter alkanes (C_4_-C_11_) [117]. Species belonging to the aforementioned genera have often been isolated from crude-oil-contaminated matrices [118,119,120] and successfully used in bioaugmentation applications. For instance, the *Candida tropicalis* SK1 strain was able to remove 83% of aliphatic hydrocarbons from a clay–loam soil with pollutant load of 16,300 mg/kg after four months’ treatment [121]. The only amendment used was a mineral solution made of ammonium sulfate and KH_2_PO_4_ to yield a soil C:N:P ratio equal to 100:10:5. The augmentation of a diesel-contaminated soil mixed with food waste (77:23 *w*/*w*) (initial C_10_-C_28_ aliphatic hydrocarbon content, 10,189 mg/kg) with the *Candida catenulata* CM1 strain attained a 84% removal after 13 days, with a significant reduction of alkanes [122].

Another group of hydrocarbonoclastic Ascomycetes includes black yeast-like fungi (BYLF), which includes the two neatly distinct orders of Dothideales and Chaetothyriales. This group owes its name to the dark color of their cell walls, due to the presence of melanin, which, along with other protective substances such as mycosporines and carotenoids, gives BYLF the ability to colonize extreme environments [85]. BYLFs are well known for their efficient degradation of alkylbenzenes [123], but some species belonging to the genera *Phialophora*, *Aureobasium*, *Exophiala*, and *Rhinocladiella* can also degrade APH. For instance, April [124] found a *Phialophora* isolate able to degrade the C_12_-C_26_ aliphatic fraction of crude oil.

Within the phylum Basidiomycota, the frequently detected presence of several hydrocarbonoclastic species belonging to the group of basidiomycetous yeasts in crude oil-contaminated soils has suggested their potential use in bioremediation [119,125]. These species belong to the genera *Rhodotorula*, *Rhodosporidium*, *Cryptococcus,* and *Trichosporon*, and although their ability to degrade aliphatic hydrocarbons has been proven by several studies [120,126]; there are very few documented examples of bioaugmentation of crude-oil-contaminated sites using these organisms.

By contrast, attention has been focused on either wood- or litter-decomposing species belonging to the subphylum Agaricomycotina and the orders of Agaricales and Polyporales, which share the possession of an efficient lignin-degrading machinery operating in the extracellular environment via radical-based reactions [97]. Although most remediation studies with these fungi have targeted aromatic pollutants (e.g., chlorophenols, PAHs, polychlorobiphenyls), due to their structural resemblance with some lignin substructure (e.g., alkyl-arene, catechol diethers), significant efforts have been made to evaluate their performance in crude-oil-contaminated soils. Among these species, a variety of bioaugmentation studies have been conducted with *Pleurotus ostreatus* on soils artificially spiked with either crude oil or refined products [127,128,129], although the contribution made by this species to the removal of alkanes remains controversial. In fact, Pozdniakova [128] suggested the need for synergism between *P. ostreatus* and the indigenous microbiota in the degradation of APH, while [127] showed that this species, albeit with low efficiencies towards long-chain alkanes, depleted these contaminants from a soil that had been previously sterilized. Covino [36] found that bioaugmentation of a historically contaminated and fine-textured soil with *P. ostreatus* resulted in an 86% APH removal after 60 days’ incubation; the same study also highlighted that the residual contaminant concentration was lower than the non-bioavailable fraction, estimated by a sequential supercritical fluid extraction under mild conditions. An interesting study comparing the efficiencies of several bioremediation approaches applied to a highly hydrocarbon-impacted soil (initial C_10_-C_40_ hydrocarbon content, 340 g/kg) showed that the best results (86% PH removal after 90 days’ treatment) were obtained with *P. ostreatus* inocula, where the lignocellulosic amendment was partially mixed with soil and partially layered on top [130]. An undoubted advantage of using white-rot basidiomycetes is that this ecological group includes several edible species, such as *P. ostreatus*, *Lentinula edodes*, *A. bisporus,* and *Ganoderma lucidum*. This means that their residues from the production of carpophores, called spent mushroom substrate (SMS), can be used as readily available inocula, and this opportunity has been seized. For example, inoculating historically PH-contaminated soil with 10% SMS of *A. bisporus*, *P. ostreatus*, and *G. lucidum* resulted in PH removal levels of 71.5, 69.5, and 57.7%, respectively, after three months of incubation [131].

Despite the great capabilities shown by ligninolytic fungi, their exploitation for bioremediation has several bottlenecks: (i) moderate and species-dependent adaptability to soil conditions (e.g., pH, moisture); (ii) need for lignocellulosic material for their growth, (iii) moderate ability to compete with the autochthonous microbial community; and (iv) accumulation of toxic degradation intermediates.

Other types of fungi have been indicated as exploitable in this field, such as litter-decomposing fungi, which are able to degrade contaminants and adaptable to soil environments. Their presence also enhances the activity of autochthonous fungal and bacterial communities. The latter are particularly important in the process of bioremediation, and sometimes a biostimulation approach is enough to achieve good results in terms of removal efficiency. Biostimulation or bioaugmentation and biostimulation by using compost or autochthonous isolated fungi can be applied with great success, exerting a positive impact on the microbial community itself. In the bioremediation field, interactions with the autochthonous microbial community are pivotal, as will be shown in Section 2.4 of this review.

#### 2.2.2. Fungal Metabolic Pathways

**Figure 1:**
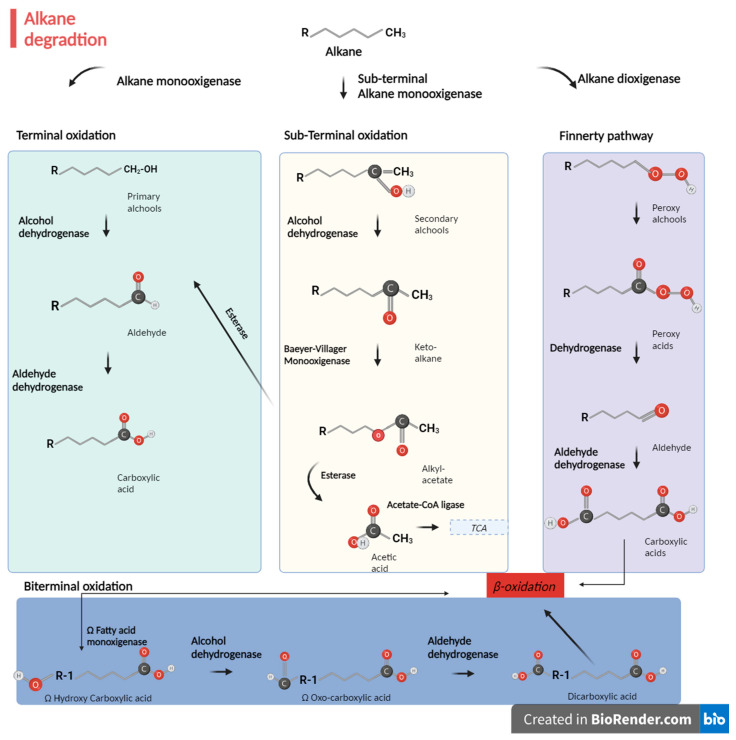
Possible pathways of the aerobic degradation of *n*-alkanes by fungi. Bifurcation is possible at the end of terminal oxidation pathway: the obtained carboxylic acid can either go through β-oxidation or be further oxidized by the ω-fatty acid monooxygenases to form dicarboxylic acid (biterminal oxidation). The products of the sub-terminal oxidation pathway are secondary alcohols or methyl acetone, which can be further oxidized by Baeyer–Villiger monooxygenases and esterases to generate fatty acids and primary alcohols. The first step of the Finnerty pathway is the formation of *n*-alkyl hydroperoxides by alkane dioxygenases that are oxidated to fatty acids [141]. Figure created with BioRender.com.

For aliphatic assimilation, the main degradation route is intracellular in microsomes, and it is initiated by oxygenases such as cytochrome P450 (CYP) monooxygenases that introduce oxygen atoms into *n*-alkanes [132]. Cytochromes are the terminal oxidases in a chain of electron transfer catalysis with NADPH reductases, which supply electrons to insert oxygen in alkanes while reducing oxygen to water [133]. After the first oxidation to primary alcohol, the alkanes are transformed into aldehydes and finally into fatty acids. This is the most common pathway, although there are another four possibilities, listed and described below (also summarized in Figure 1):

- The terminal oxidation pathway [134] is involved in the oxidation of the terminal methyl group of *n*-alkanes. The initial product of the reaction is a primary alcohol, which is sequentially oxidized by alcohol dehydrogenases and aldehyde dehydrogenases to a fatty acid that enters in β-oxidation [135]. This is the most common pathway, as already mentioned.
- During the biterminal oxidation pathway, both terminal methyl groups of the *n*-alkane undergo oxidation to the corresponding fatty acid without breaking the carbon chain. The product of the reaction is a ω-hydroxy fatty acid, further converted to a dicarboxylic acid, entering in β-oxidation [135,136,137].
- Subterminal oxidation has been also observed to form primary alcohols and secondary alcohols or methyl acetone with the same chain length as the substrate [138].
- During the *n*-alkyl hydroperoxide pathway in *Acinetobacter* sp. strain HO1-N [139], the *n*-alkanes are oxidized to peroxy alcohols and then to peroxy acids, alkyl aldehydes, and, finally, fatty acids [139]. A dioxygenase is responsible for the first step of oxidation [140]. This pathway is the least common.

Aliphatics, such as branched chain aliphatics or alicyclic aliphatics, can be oxidized by fungi and yield as intermediates epoxides, alcohols, diols, and carboxylic acid units, which generally are not used as growth substrate, and alicyclics are also more recalcitrant to biodegradation than aliphatics [142]. There are few examples in the literature regarding fungi able to grow on alicyclic aliphatics, such as anamorphs of *Ophiostoma*. Some yeast species belonging to the genera *Candida* and *Trichosporon* were reported to be able to transform cyclohexane to cyclohexanone [143]. Notably, the black yeast *Exophiala jeanselmei* is able to use cyclohexanone as the sole carbon source [144]. Although there is little information on the degradation pathways of alicyclic aliphatics, it is pretty clear that the complete degradation of these contaminants requires synergism between microbial community members.

### 2.3. Bulking Agent and Formulation of the Inoculum

The remediation of polluted soil is a melting point of different parameters that can achieve great results if mixed well together. Considering soil texture, autochthonous microbial community, and nutrient and water contents, one of the ways to combine all these parameters is a bulking agent.

A bulking agent is defined as a material that provides the optimum free air space (FAS) and regulates the water contents of the waste to be composted. Bulking agents are commonly fibrous and carbonaceous materials with low moisture content to provide optimal FAS for the composting process [145,146]. Alternatively, inorganic bulking agents are available, such as expanded clays or mineral foams (rockwool or glasswool), to avoid an increase in the carbon content of the system. Bulking agents give structure, decrease bulk density, increase porosity in the matrix (microporosity, in particular), maintain moisture, increase the amount of macronutrients, reduce the improper balance between C and N, and reduce hydrophobicity. For this purpose, agro-industrial wastes can be used since they are a slow-release source of nutrients, such as phosphorus, nitrogen, potassium, and carbohydrates.

Different types of bulking agent and inoculation can be applied depending on the soil’s texture. Bulking agents can modulate aeration and moisture in the matrix [147]. In a soil rich in clay particles, a higher aeration effect is appropriate, while in sandy soil, where the moisture is held with difficulty, it is better to use a bulking agent that keeps the water in place. In this case, sawdust, rice husk, cotton waste, and maize straw can control the moisture content of the soil. Meanwhile, sawdust, rice bran, rice husk, cotton waste, maize straw, and peanut shells can modulate aeration and create free air space.

The choice of a bulking agent is important in the mycoremediation process and in bioremediation in general. The combined ability of bulking agents to provide nutrients and modulate aeration and moisture can lead to the biostimulation of the autochthonous microbial community. Also, a lignocellulosic material, used as a bulking agent, can help the inoculum, triggering the production of enzymes. Moreover, the bulking agent addition needs to be taken into account in the total calculation of nutrients to avoid an excess of organic matter added to the matrix and thus its potential prioritized degradation instead of the contaminants.

Regardless of the general action performed by bulking agents in bioremediation, an appropriate choice of these agents is fundamental in the case of mycoremediation especially in the case of WRF. In fact, Meysami [148] showed how different strains of WRF were unable to colonize soil contaminated with hydrocarbons in the absence of a bulking agent. The same study showed how mixing two bulking agents, such as peat moss and bran flakes, with different physicochemical characteristics, allowed faster growth of the investigated strains. Among different bulking agents (i.e., rice husk, vermicompost, sugarcane bagasse, and coconut coir), a binary fungal–bacterial consortium performed the best alkane and TPH removal (95 and 82%, respectively) from a soil spiked with 2% crude oil in the presence of rice husk and a ternary fertilizer after 30 days of incubation [149].

A further factor that should not be overlooked is the role played by bulking agents as valuable carriers of the inocula of saprophytic fungal species. Unlike bacteria, for instance, the ecological group of WRF cannot generally use organic pollutants as C and N sources and, thus, an external nutritional supply is needed [97]. In this regard, natural carriers, such as lignocellulosic materials, provide mycelia with nutrients, thereby giving them a competitive advantage as compared to free inocula [150,151].

In mycoremediation, the bioaugmentation with fungi is pivotal. As discussed above, the fungal species exploitable for mycoremediation belong to different divisions and phyla, and so they belong to different ecological niches and have different growth/production as well. In the case of WRF, bioaugmentation can be inoculated in the soil by the addition of agro-waste inoculated with the determined fungal species and act as a bulking agent as well. As described previously, the main WRF ecological niche is wood material, so growth and enzyme production are supposed to occur in the presence of wood material. In the case of Ascomycota, the inoculation can occur in different ways, either by spores or by liquid inoculation. A prior assessment of the two methodologies might help to discern the best way for mycoremediation purposes.

The amount of inoculum media is also important for mycoremediation. A wide range of inoculation ratios, from 3% up to 20%, has been reported (Table 3). Taking into account the scaling up of the technology, the amount of inoculum media should be the minimum possible in order to reduce the costs and energy impact. The use of WRF is convenient since it generally recycles agro-waste, thereby decreasing costs and enhancing the circular economy. From the calculations performed in previous studies by Chicca [152], a 10% inoculation media with *Ciboria* sp. (Ascomycota) is not sustainable in terms of costs, using beer malt waste in a rich medium instead of commercial malt extract for the scaling up. While using a 1% inoculation rate, the overall process was more sustainable. In addition, the exploitation of WRF using agro-waste is more sustainable, but if a bulking agent is also used, the balance of nutrients should be considered, as discussed below.

**Figure 2:**
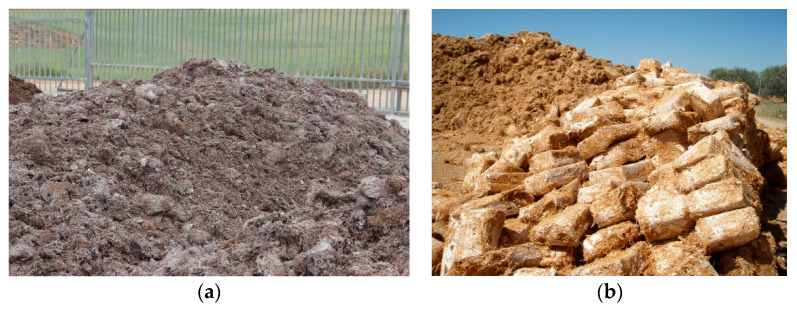
Pictures of spent mushrom substrate (SMS) of *A. bisporus* (**a**) and *P. ostreatus* (**b**).

A popular and cost-effective bulking agent that can be used in soil mycoremediation is spent mushroom substrate (SMS). This material is the by-product of edible mushroom cultivation. SMS consists of partially degraded lignocellulosic materials (e.g., straw, wood chips, sawdust, corn cob, etc.), other organic by-products (e.g., livestock litter, manure, grape marc, etc.), amendments (gypsum, calcium carbonate, peat), and the residual fungal mycelium. It is important to note the wide variety of substrate formulations based on the mushroom cultivated and the location and availability of lignocellulosic wastes and organic by-products of the region, among other factors. Hence, the different SMS have variable physico-chemical and biological characteristics, as shown for SMS of *A. bisporus* and *P. ostreatus*, two of the most widely cultivated mushrooms around the world (Figure 2).

World production in 2017 of edible mushrooms was around 10.2 million tons, of which there are two species that are produced the most: *A. bisporus*, 15%; and *P. ostreatus* mushroom, 19%, with China being the largest producer and world exporter of these two species [156]. In the European Union, there are three major producers of mushrooms: the Netherlands, France, and Spain, which account for 51% of production and, therefore, generate localized environmental problems [157].

SMS is rich in organic matter and nutrients; large volumes are produced, and it ends up becoming waste [156]. The substrate of *A. bisporus* is compost produced by wheat straw and manure (litter manure from cows or horses). By the end of the crop cycle of *A. bisporus*, the content of lignin, cellulose, and hemicellulose is metabolized at almost 50%. However, the content of the forms of carbon are remarkable: 8.6% of lignin, 6.3% of hemicellulose, and 12.0% of cellulose, respectively [158]. Regarding *P. ostreatus*, its substrate is mainly fermented straw [159]. Each kg of caropophores produces between 2.5 and 5 kg of SMS [160]. This waste causes an environmental problem due to its disposal in landfills, which generates leachates affecting groundwater. In addition, this waste is also deliberately burned, thus affecting the atmosphere by generating greenhouse gases and the greenhouse effect [156]. The wide extensive possibilities to re-use SMS, alleviate its environmental issues, and promote the circular economy, include references to the application of SMS in soil bioremediation as a bulking agent and/or inoculum medium to mycoremediate TPH and other organic pollutants [131,154,161,162]. In this context, SMS serves as an inoculum source, enhances soil water retention capacity, provides nutrients to soil microbiota, and stimulates soil microbial biomass and activity, with the consequent increment of pollutants degradation.

SMS also contains residual enzymes such as cellulases, hemicellulases, proteases, lignin peroxidase, manganese peroxidase and laccase that are still useful, as the efficiency of these enzymes has been proven to degrade PAH, pesticides, chlorinated phenols, and other compounds of environmental concern [163].

### 2.4. Bacteria–Fungi Interaction in Mycoremediation

In nature, bacteria and fungi often share microhabitats, forming complex communities, also known as the microbiome. Bacteria and fungi interactions (BFI) play a key role in the functioning of numerous ecosystems: they are cornerstone members of communities driving biochemical cycles [164,165].

BFI intrinsically modulate the behavior of either or both of the interacting partners. Such modulation cannot be easily predicted based only on the knowledge of the biology of the isolated microorganisms grown in pure cultures. Within the past decade, a range of multidisciplinary studies on diverse BFI, which integrate tools from molecular biology, genomics, chemical and microbial ecology, biophysics and ecological modeling, have emerged [164].

Within bioremediation, which involves co-metabolism and a hierarchical relationship of contaminant degradation among many microbial communities, there are many expectations for fungi and bacteria to remove toxic recalcitrant compounds in a sustainable and synergistic manner.

The synergy between the fungal and bacterial community in the soil framework might include physical interactions via dispersal of degradative bacteria by fungal hyphae, and chemical interactions via degradation of organic contaminants, their co-metabolism, and enhancement of degradative enzyme production and secondary metabolites [10].

#### 2.4.1. Physical Interactions

Fungal growth is supported by apical growth of hyphae, which can disperse in soil through pores and across air gaps, while, thanks to the aid of hydrophobin production (amphiphilic proteins) [166], leading to a reduction in surface tension at the interfaces, a higher solubilization of hydrocarbons is allowed. Fungal growth can also occur in unsaturated pores [43].

Kohlmeier [167] demonstrated that the mycelial network can act as “fungal highways […] on which bacteria can move actively in spaces where they can find substrate to grow on”. Mycelial networks acting as ‘highways’ would allow chemotactic bacteria to swim or swarm, overcoming motility restrictions thanks to the presence of a thin film of water on the hyphal surface, and reach remote areas [168]. The transportation, in fact, depends on the bacteria themselves, and their mobility skills, and on the characteristics of the fungal products, such as mucoid liquid films around the fungal hyphae. Fungi, moreover, can produce exudates which bacteria can be fed onto.

Whatever the mechanism, mycelial networks represent an opportunity to increase the frequency of contact between hydrocarbonoclastic bacteria and hydrophobic contaminants, thus mitigating problems related to the low bioavailability of contaminants [169]. In general, mycelial networks provide a very robust infrastructure, reducing the time required to degrade pollutants under various abiotic conditions typical for water-unsaturated soils.

Several methods have been developed for the investigation and isolation of bacteria dispersed through fungal networks [170,171], including for hydrocarbon degrading bacteria using fungal mycelia [172]. Studies on BFI have demonstrated that mycelia-facilitated bacterial dispersal may likewise promote new niche colonization [173,174,175] and contribute to bacterial food spoilage [176] or the co-invasion of tissues during pathogenesis [177,178]. However, only a few studies were dedicated to PH degradation.

Another physical way in which fungi can help and synergistically work with bacteria is presented by “fungal pipelines”, where molecules could be taken up by simple diffusion or by active translocation into the mycelia. This mechanism allows the transportation of nutrients as well as pollutants along the fungal hyphae. Fungal pipelines are demonstrated to be facilitators for bacterial degradation of pollutants by bringing them from remote areas through the mycelia [168,172].

Whether highways or pipelines are active or are the predominant mechanism in remediation depends on the bacterial state: while highways seem to be related to active/mobile bacteria, pipelines are more relevant with inactive starving bacteria. Together, these mechanisms develop a robust system that improves degradation, especially in low water activity environments [168].

#### 2.4.2. Chemical Interactions

Fungi and bacteria have different approaches to exploiting new nutrient sources, including organic contaminants. Fungi in general, and saprotrophic basidiomycetes in particular, are efficient degraders of recalcitrant organic compounds (lignin and plant cell wall polysaccharides) while bacteria in soil are more successful in the decomposition of simple substrates [179,180]. Due to different preferences for substrate, fungi and bacteria occupy different niches of pollutant degradation. Filamentous fungi, as K-strategists, have better individual qualities and preeminent resistance to harsh environments [181]. They have a stable growth rate and spend more energy on ectoenzyme production and defending against severe environments, which enables them to degrade more complex and toxic organic compounds [182] including through co-metabolism reactions. By contrast, specialist bacterial species are expected to be responsible for the transformation of contaminants in polluted environments, since they can utilize them as a carbon source [183].

For instance, the degradation of high-molecular-weight compounds such as long-chain alkanes or PAH can be challenging for microbes, given their poor solubility in water. Thus, the cooperation of fungi, which can oxidize these compounds to metabolites with higher aqueous solubility by secreting their extracellular enzymes, and bacteria that can mineralize the metabolites is advantageous [80,184,185].

#### 2.4.3. Synergic and Antagonistic Interactions

Microbial interactions, in general and especially in complex matrices such as the soil, can be very difficult to predict, and, at the same time, pivotal for the overall process. Bacterial and fungal communities, especially if one of them is allochthonous, may interact in a synergistic and/or antagonistic manner.

Several examples of positive interaction between bacteria and fungi have been reported which suggest that the reinoculation of specific co-cultures of fungi and bacteria in lab-scale biopiles enhances biodegradation of light and heavy fractions of aliphatic hydrocarbons compared with that of respective pure cultures (*Acremonium* sp. and *B. subtilis*) [186].

Liu [187] performed orthogonal experiments to study the effect of different factors for the optimization of the bacteria–WRF joint remediation system. The highest degradation rate for PH (57%) was achieved after 30 days of incubation by the combination of bacterial culture (*B. licheniformis*) and fungal strain (*P. ostreatus*) grown in solid-state fermentation with the addition of sand, straw, and biosurfactants. The better performances were possibly due to the fungal enzymes released in soil which degrade complex petroleum hydrocarbons and metabolites then utilized by bacteria/ microorganisms in soil.

Given the fact that soil is not their ecological niche, WRF were inoculated and persistent during the entire bioremediation experiment for 200 days, while, in other cases, the addition of WRF and substrate did not make any significant difference in biodegradation efficiency compared to the addition of the sole substrate [183]. Equally, in some other cases, the inoculated strain might be active in the first phase and decrease during the process.

In this frame, SMS may represent a low-cost strategy to provide valuable inoculum for the mycoremediation process, in order to supply the polluted soil with WRF and their related enzymes, and the specific bacterial community enhancing contaminant degradation [154]. Different SMS types of four fungal species (*Pleurotus eryngii*, *Lentinula edodes*, *Pleurotus ostreatus,* and *Agaricus bisporus*) were inoculated in a PH-polluted soil [154]. The results showed that *Agaricus bisporus* SMS inoculation achieved 48% of APH depletion after 40 days, leading to the hypothesis that the applied SMS stimulated the autochthonous microbial community of the soil to accomplish PH degradation, supported also by the significantly higher activities for hydrolases and dehydrogenases enzymes compared with the control. Similarly, Zhou [188] found that the utilization of SMS with *Pleorotus eryngii* led to the stimulation of the autochthonous bacterial community, such as *Microbacterium*, *Rhizobium,* and *Pseudomonas*, which are able to degrade PAH. Conversely, Becarelli [189] compared two different approaches based on the utilization of a SMS and autochthonous *Lambertella* strain augmentation to treat PH-contaminated dredged sediments. After 28 days, in the *Lambertella-*augmented microcosms, PH depletion was almost complete, while there was no PH removal in SMS-augmented ones after 60 days. In this case, SMS did not help to enhance PH degradation, not as a result of competition but because it prioritized the transformation of organic matter, while *Lambertella* achieved the high depletion rate due to its ability to mobilize the contaminants given its saprophytic profile and its adaptation to that niche.

Although several studies report synergistic effects of FBI in bioremediation, they may have competitive behavior. The mechanisms leading to the suppression of bacteria by fungi are not clear, and what makes a particular species a weak or a strong competitor is unresolved [180]; in all cases, ligninolytic basidiomycetes such as *Pleroutus*, *Phanerochaete,* and *Trametes* are reported to be strong competitors.

Some examples of competitive behavior are the production of phenazine derivatives, 2,6-diacetylphloro-glucinol, antifungal antibiotics by bacteria towards WRF [190,191], or mycophagy [179]. Otherwise, WRF can also hamper the growth of soil bacteria by lysing cells to limit the exploitation of easily degradable metabolites from lignocellulosic substrate. Ligninolytic enzymes might be used as a mechanism of passive defense by the formation of melanin or similar compounds, or in an active defense by producing oxidizing molecules [180]. This mechanism is also evidenced in experiments carried out by Liu [187] where the mutual effect between laccase and the *B. licheniformis* Y-1 used in the degradation of petroleum hydrocarbons was investigated. Laccases of *P. ostreatus* seem to inhibit bacterial growth of the co-inoculated *B. lichenifomis*.

#### 2.4.4. Metagenomics as Tool to Study Microbial Interaction in Bioremediation

The spectrum of microbial interaction in bioremediation may be very wide, making it uncertain as treatment. The study of the interactions and the population at the molecular level might be a powerful tool to promote bioremediation efficiency and clarify dynamics among bacterial microbiota and inoculated fungi. The advent of high-throughput sequencing technology has revolutionized the study of environmental microbiology [192]. Next-generation sequencing (NGS) methods allow to study the entire community of microorganisms inhabiting an environment. They provide an opportunity to explore unculturable microorganisms.

Several studies aim to describe bacterial and fungal dynamics in different PH-polluted soils in bioremediation processes using metagenomic approaches [141,193,194]. In general, it seems that bacterial communities changed drastically, showing different successions in time, which was mostly due to the harboring of specific enzyme complexes that can degrade hydrocarbons. Fungal communities showed less significant changes than the ones observed in bacterial communities, mainly due to the non-specific enzymes enabling them to degrade lignin, hemicelluloses, and aromatic compounds [193]. These results are consistent with the evidence reported by Geng [194] that PH contamination has a greater impact on the bacterial community structure being more sensitive and responsive to PH than the fungal community.

More specifically, it seems that Proteobacteria and Bacteroidetes had higher adaptability in soils contaminated by high PH concentrations, while it has been hypothesized that PH-polluted soil may inhibit Ascomycetes [194]. It was additionally demonstrated that the increase in the PH contamination level decreased the network connectivity (i.e., the network degree) of the bacterial and fungal taxa. Specifically, the number of network edges of the bacterial taxa in the heavily PH-contaminated soils was reduced by 29% compared with the lightly PH-contaminated soils [194]. Further research would be necessary to discover the mechanisms involved.

Covino [36] showed that a soil with an aged PH contamination containing 10,000 mg/kg of aliphatic petroleum hydrocarbons was significantly depolluted by bioaugmentation with *P. ostreatus* CCBAS278, the ITS amplicons of which had a relative abundance of 60% after 60 days incubation. In the *P. ostreatus*-augmented microcosms, the relative abundances of Gram-positive taxa, in particular Actinobacteria, were significantly lower than those in the non-augmented incubation control. In the same study, when the soil was bioaugmented with *Botryosphaeria rhodina* DABAC P82, the bacterial community structure was very similar to the non-inoculated incubation control, presumably due to the very low persistence of the applied inoculum [36].

Medaura [112] conducted a microcosm experiment to study the degradation of aliphatics and aromatics by biostimulation and by bioaugmentation. The addition of autochthonous fungi (*Penicillium* spp., *Penicillium chrysogenum*, *Ulocladium* spp., *Ulocladium atrum*, *Aspergillus terreus*, *Fusarium oxysporum*, and *Aspergillus parasiticus*) showed a diversification of the bacterial community: there were some species common at the three treatments (one was the control), other species appearing just in biostimulation and bioaugmentation (*Promicromonospora* sp., *Olivibacter* sp., *Sphingopyxis* sp.), and others just in bioaugmented microcosms (*Streptomyces* sp., *Azocarus* sp., *Lascolabacillus* sp., *Fermentimonas* sp.). In some cases, undescribed species belonging to the orders Cytophagales, Bacteroidales, and Rhodocyclales, which have never been previously observed in hydrocarbon-polluted sites through molecular methods, were discovered. This finding provides a first insight into the dimension of the as-yet-unknown biodiversity and interactions between hydrocarbonoclastic microbial communities. The inoculated species were fast growers and ubiquitous, but, at the same time, were reported to be able to degrade completely most fractions of aliphatic hydrocarbons, up to C_30_, in the polluted soil.

Becarelli [183] studied the succession of bacteria and fungi in a microcosm experiment where biostimulation and bioaugmentation approaches were applied to treat a PH-polluted soil for 90 days. The bioaugmentation approach was applied with two different inoculation ratios (7% and 1%) of a *Ciboria* sp. Strain, and the results were different in different stages of the process, but they followed similar behavior. In fact, in both cases, it was possible to observe a succession of different bacterial species presumably involved in the different stages of the process. At first, saprotrophic microbes, either bacteria or fungi, such as *Arthroobacter*, *Dietzia*, *Brachybacterium*, *Brevibacterium*, *Gordonia*, *Leucobacter*, *Lysobacter*, and *Agrobacterium* were detected in significant percentages and their presence was correlated with Dye peroxidase. Meanwhile, in a second phase, corresponding to the highest PH depletion, a community of PH degraders such as *Streptomyces*, *Nocardoides*, *Pseudonocardia*, *Solirubacter*, *Parvibaculum*, *Rhodanobacter*, *Luteiomonas*, *Planomicrobium*, and *Bacillus* spp., correlated with hydrocarbon degradation functions, substituted the first one. At the end of the experiment, the microbial population was more balanced. Thus, a succession of what can be defined as generalist microbes, harboring non-specific enzymes involved in the oxidation/mobilization of organic matter and contaminants, and specialist microbes, harboring enzymes involved in PH depletion, was observed in both the bioaugmentation ratios tests. The inoculated strain, *Ciboria* sp. was detected at the beginning and showed an increase at the end of the process and not during the phase of highest PH depletion, leading to the hypothesis that the inoculation was pivotal in the first stage to prime the overall process.

In order to transform the soil mycoremediation process into a robust technology, a comprehensive understanding of the physiology, ecology, and phylogeny of the microbial interaction between autochthonous microbiota and fungal inocula is essential. However, most studies on ecological processes, especially investigations on bioremediation, concentrate on the analysis of microbial community composition based on the 16S rRNA gene, omitting its functional and metabolic properties [195]. The predictive functional metagenomics should be considered as the basis for developing a robust predictive instrument to infer the functions that the microbial communities and colonizing contaminated matrices can express and, consequently, the functions to be exploited to complete and even accelerate a decontamination process [141].

However, besides the presence of several studies focusing on predictive bacterial functionality in PH degradation [183,196,197], studies reporting predictive fungal functionality on PH degradation are still missing, although some platforms such as FUNguild [198] have recently appeared.

### 2.5. Environmental Factors Affecting Mycoremediation, Soil Properties

Mineral soil can be a poor source of available carbohydrate; yet, despite this, many fungi can maintain growth in nutrient-limited habitats. It has been suggested that these organisms possess characteristics that enable them to utilize low nutrient supplies efficiently, including an increased capacity to take up nutrients by possessing a high surface area resulting from sparse but extensive mycelium, high affinity nutrient uptake sites, and translocation of nutrients from a nutrient-rich base. Germ tubes and hyphae may be reduced in diameter and length under carbon-rich conditions [199]. On the one hand, contaminants can be used as nutrients by microorganisms or degraded by co-metabolism in the presence of other carbon sources. On the other hand, the presence of a high concentration of hydrocarbons alters the physicochemical and biological properties of the soil, such as cation exchange capacity, hydrophobicity, and electrical conductivity, preventing gas exchange with the atmosphere [147]. These alterations also affect the native microbiota. Nevertheless, PH can be toxic towards microorganisms and human and environmental health, and accumulate in the environment by absorbing to organic matter in the food chain. This is especially true when the soil is historically contaminated due to the sorption of organic contaminants to solid surfaces, by being trapped physically in the micropores and absorption to organic matter [7].

The rates of biodegradation of organic compounds in soil and groundwater environments have been shown to be controlled by the rates of mass transfer from sorbed phase or nonaqueous phase liquid (NAPL) to the aqueous phase or by their intrinsic rates of biodegradation. Petroleum NAPLs discharged into soils can migrate into micropores of soils by altering the wettability of soil mineral domains through a multistep process, which starts with binding of the polar fractions of oil to soil mineral surfaces. In addition to pore size exclusion, irreversible sorption to soil background organic matter (glassy organic matter) may contribute to limited bioaccessibility and the bioremediation endpoint of hydrophobic compounds. In the case of fine-grained, clayey soils, where a significant number of pores are smaller than or equal to the range of bacterial aggregate size, and thus direct contact between entrapped oil in small micropores and bacteria would not be feasible, the bioaccessibility of a fraction of poorly soluble hydrocarbons will likely be limited. However, considering the significant volume of bioaccessible pores, a significant biodegradation extent is potentially possible. In coarse-grained, less porous soils, where pores are predominantly bioaccessible, the bioremediation endpoint will be mainly governed by the biodegradability of residual NAPL components [200].

Spilled contaminants enter into soil pores and adsorb onto soil particles, moving vertically with capillary and gravitational forces, which alter their chemical, physical and biological properties and composition [201].

Compared with bacteria, filamentous fungi show some advantages in the transport or translocation of essential substances, as mentioned before, including nutrients and water, and the pollutant itself, over significant distances [202]. The ascomycetes strain, *F. neocosmosporiellum*, with biosurfactant production ability and positive laccase activity can degrade approximately 90% of crude oil in PDB at 28 °C. Also, this strain can degrade about 40% of crude oil under optimum conditions of the soil microcosm. Optimization of the condition indicated that the C:N:P ratio of 100:10:1 is the optimal ratio for contaminants removal from the soil [203]. The C:N:P ratio is a pivotal parameter, discussed in depth later.

Several soil parameters have a strong influence on bioremediation. The optimum conditions for oil degradation have been proposed in the literature, namely: soil moisture 50–60% (soil should be wet but not puddly), soil pH 6.5–8.0, nutrient content C:N:P = 100:10:1, temperature 20–30 °C, and hydrocarbon concentration 5–10% of the dry weight of soil and with low clay or silt content (minimum air filled pore space of 10–40%) [147]. The following factors should be considered since they significantly affect the biodegradation process:

- Soil texture: This can influence the remediation of the soil both in terms of aeration and/or water-holding capacity and in terms of pollutant concentration. Depending on soil texture, the transition of oxygen, nutrients, and water to the zone of biological activity might change. The fine particles of soil, such as silt and clay, transport these elements slowly. Permeable soils, which contain gravel and sand, are suitable for nutrient transmission and can be treated relatively quickly. The addition of a bulking agent can be helpful, on one hand to increase the porosity in clay soils, and thus the aeration to ensure the oxidative reactions, while on the other hand, it can help to increase the capacity to retain water in sandy soil. Fungal biodegradation of hydrocarbons is led by an aerobic process; oxygen concentration is one of the most influential speed-limiting factors, at least for the initial breakdown stages of hydrocarbon molecules [97]. Moreover, PH are vigorously and particularly adsorbed onto the clay soil particles, and desorption of these hydrocarbons from the soil is regarded as a rate-limiting factor during biodegradation. Bulking agent addition might positively change the texture of soil (see Section 2.3).
- Humidity: Aguilar-Rivera [204] reported that 70% relative humidity is ideal for mycoremediation with *P. ostreatus*. Seidu [205] reported that mushroom growth and fruiting is favored by a relative humidity of 70–80%.
- Temperature: It has been shown that mycoremediation is optimal at temperatures of 25–30 °C [206] and that the rate of degradation of organic contaminants is comparably higher at elevated temperatures [207]. Fungi involved in hydrocarbon degradation are generally mesophilic organisms, i.e., they can grow in the range 10–40 °C, showing an optimum in the range 20–35 °C [208].
- Oxygen: Given the fact that hydrocarbon degradation by fungi occurs mostly by aerobic processes, oxygen concentration is one of the most influential speed-limiting factors, at least for the initial breakdown stages of PH [97]. Oxygen levels in the soil should not be limiting to ensure aerobic biodegradation. The limiting value in terms of oxygen concentration is around 5%. The availability of oxygen in soils is dependent on rates of microbial oxygen consumption, the type of soil, whether the soil is waterlogged, and the presence of utilizable substrates, which can lead to oxygen depletion. Bulking agent addition can help in this manner in clay soil, as mentioned in Section 2.3. A proper study before starting the pilots might be carried out, in order to establish the best conditions for aeration. On the other hand, anaerobic degradation in soils has less ecological significance because it occurs only at low rates, and especially by bacteria.
- pH: a major factor in hydrocarbon biodegradation is soil acidity, because it influences enzymatic activities, cell membrane transport and catalytic reaction balance [209]. It also affects microbial growth; it can influence the fungal–bacterial relationship by promoting or inhibiting the growth of one of the partners, and it can also influence the fungal hyphae-mediated migration of bacteria. At pH < 5.0, bacterial growth rate was slow and fungal growth was promoted, whereas in higher-pH soils (pH 6.5–8.0), low fungal growth rate was observed while bacterial growth increased. When bacterial growth was suppressed, increased fungal growth was observed even in high pH soils, which suggests that bacteria were causing competitive pressure inhibiting fungal growth at high pH [10]. Different fungi have different pH preferences for optimal growth and activity [210]. Some species thrive in acidic conditions, while others prefer alkaline or neutral pH levels. For this reason, it is crucial to select fungi that are well-adapted to the pH range of the contaminated soil for the best results in myco-augmentation. Moreover, pH affects both production and activity of extracellular enzymes by directly acting on the oxy-functionalization of alkanes, such as unspecific peroxygenase [211], or indirectly via the mechanism of the quinone redox cycle, such as in the case of laccase [53]. Furthermore, some nutrients, phosphorus in particular, may become available to fungi depending on the pH level. Finally, the pH level can influence the composition and diversity of microbial communities, including the presence of potential competitors or mutualistic organisms that might affect the mycoremediation process.
- Nutrients: The balance of macronutrients is pivotal for remediation purposes; in fact, generally, the nutrients are balanced to reach C:N = 10. The scientific literature reported a different number of C:N ratios. Marion [212] highlighted optimal C: N ratios between 11 and 27 [on the weight basis]. Among the different examples, it is possible to find Venosa [213], who obtained a PH removal efficiency of 90% for alkanes for a C:N:P ratio of 150:10:3, while Sanscartier [214] used a C:N:P ratio of 100:7.5:0.5 to obtain a removal efficiency of 90–99% of different alkane fractions (C:N ratio at 15 for both). The study by Grace Liu [215] consisted of the use of three different C:N:P ratios: 100:27:6.5, 100:11:3.7, and 100:4.6:3.1. Successful stimulation of the communities was achieved, with 100:11:3.7 corresponding to APH removal of 85–95%; this ratio was close to the nutrients required for the recommended C:N:P ratio [100:10:1] for biopile operation [USEPA, 2002] and also the desired ratio [100:15:1] for ex situ bioremediation [216]. Ouriache [217] conducted a study comparing two different C:N ratios: 100:10 and 60:2, for the PH remediation, achieving at the end of five weeks a PH depletion of 62% for the C:N ratio 10. In the first two weeks, there was no PH depletion, but the microbial population registered an increase in CFU, given by the exploitation of organic matter. After the first two weeks, the two conditions registered an increase in C:N ratio to 17 and to 44 for conditions C:N 10 and 30, respectively. In the second case, the C:N:P ratio increased up to a level between 88:2:1 and 537.5:1.3:1. Thus, the microbial growth would be the result of the used substrate in organic matter in this case. The balance of macronutrients is pivotal to avoid the prioritizing of the organic matter over PH degradation. The nutrients must be weighted, in order to ensure growth and not prevent biodegradation by adding too much organic matter. There is a lack of information about the optimal chemical species (nitrate, ammonium, urea) to increase the level of N in contaminated soil. When adding bulking agents, which must be taken into consideration in nutrients balance, ones with a slow release of nutrients are preferred. Moreover, nutrient addition due to inoculum supply should be taken into account for the nutrient balance, especially when the amount of added inoculum media is high.
- Organic carbon: In general, higher soil organic carbon (SOC) leads to lower bioavailability of PH, since soil with higher SOC content has a higher sorption capacity for PH. Sandy soils tend to have lower amount of SOC, but the solubility of the organic matter is an important parameter. SOC decreases the availability of TPHs.
- Hydrocarbons: The biodegradability of PH can be ranked as: linear alkanes > branched alkanes > low-molecular-weight alkyl aromatics > monoaromatics > cyclic alkanes > polyaromatics > asphaltenes [218]. They differ in their susceptibility to microbial attack, and they have generally been ranked in the following decreasing order: *n*-alkanes > branched alkanes > low-molecular-weight aromatics > cyclic alkanes > high-molecular-weight aromatics and polycyclic aromatic compounds [97].

## 3. Practical Approaches and Advances in Mycoremediation

### 3.1. Soil Health Parameters

As explored until now, mycoremediation is a valuable technique for soil treatment. In general, nature-based, compared to “traditional”, physicochemical treatments are advantageous in terms of sustainability. Moreover, remediation should include not only the aim of depollution but also soil restoration in terms of functions. New regulations are highlighting the poor quality of European soil nowadays and the need for their recovery. Nature-based solutions like mycoremediation enable this by preserving the structure of soil and sustaining and enhancing naturally occurring processes.

In this frame, introducing the concept of resilience might be helpful: Resilience is the capacity of a soil, or system, to recover after a disturbance. In this case, pollution can be considered as a disturbance. Natural attenuation might measure the resilience of the soil in recovering, but it generally takes a long period of time to achieve the goal. Nature-based solutions, and especially mycoremediation, might enable and help the resilience of the soil by enhancing naturally occurring processes in the soil. Fungi are a perfect example of this. Their role in nature is to recycle organic matter by participating in the various nutrient cycles. The application of fungi might be advantageous in this respect, as it is well known that PH bonds to soil organic matter and, by transforming it, fungi might also help in the depollution purpose.

Healthy soil has been defined as “Soil with the continued capacity to function as a vital living system, within ecosystem and land-use boundaries, to sustain biological productivity, promote the quality of air and water environments, and maintain plant, animal and human health” [219]. Thus, soil health is measured through a number of parameters covering the physical, chemical, and biological nature of soil itself. Physical parameters include texture, bulk density, porosity, and aggregate stability, while chemical parameters include soil pH, electrical conductivity, cation exchange capacity (CEC), organic matter content, and nutrient levels. Finally, biological parameters include microbial biomass, microbial respiration, microbial community composition, enzymatic activity, earthworms, and nematodes. All these parameters are fully connected to each other and influence the others.

The EU regulation about soil health (currently in process of acceptance) proposed in Annex 1 a list of parameters and their relative ranges of acceptance to define whether a soil can be considered healthy or not: (1) electrical conductivity < 4 dS/m; (2) mineral soil organic carbon/clay ratio of 1/13; (3) maximum value of extractable phosphorus in the range of 30–50 mg/kg; (4) national threshold level for soil water holding capacity; (5) minimum bulk densities for the different types of soils (e.g., sandy loam < 1.8 g/cm^3^, silt clay < 1.65 g/cm^3^, and clay: <1,47 g/cm^3^; (6) soil acidity; (7) ratio of C_org_:N 1:10; and (8) soil biodiversity in the Proposal for a Directive on Soil Monitoring and Resilience of the European Commission (2023). This last parameter might be measured in different manners: (1) soil basal respiration (mm^3^ O_2_/g·h) in dry soil; (2) metabarcoding; (3) quantification of biomass; (4) quantification of macrofauna such as earthworms and nematodes.

All these parameters come together to define and assess the health of soil. The exploitation of nature-based solutions and of mycoremediation specifically can be a winning strategy for both the depollution of soil and for the recovery of the soil. For instance, bacteria and fungi are important for the formation of micro- and macro-aggregates, respectively. They are both also involved in metal weathering and in microbially-induced carbonate precipitation, wax degradation, physical entanglement, secretion of extracellular polymeric substances, and nutrient cycling, which can all mitigate soil erosion, reduce soil drought, and improve the balance of nutrients and carbon sequestration [220].

The new European regulations aim to increase the overall quality of soil health, and, even if there are many brownfields destined for industrial purposes, they cannot be left behind, and nature-based solutions might help to re-establish their resilience as well.

The interest in these techniques has risen in the recent years, and, currently, there are several European projects aimed at applying bioremediation and mycoremediation. Among the most recent are: EiCLaR (2021–2024); Nymphae (2023–2026); MIBIREM (2022–2027); BIOSYSMO (2022–2026), SYMBIOREM (2022–2026); and GREENER (2019–2023). Others have already been completed: ELECTRA (2019–2022) and Bio.Res.Nova (2015–2019). In this frame, the project LIFE-MySOIL (2021–2024) is focused entirely on mycoremediation and is designed to demonstrate and scale up mycoremediation to an industrial scale.

The application of mycoremediation at a large scale is still far from acceptance by stakeholders in the field of soil remediation (e.g., persons responsible for conducting remediation, environmental consultants, authorities). More research and applied projects might be mandatory for this scope.

### 3.2. Practical Approaches for Mycoremediation and Its Upscaling

Scaling up is a pivotal step for the exploitation of an innovative technique for soil remediation in the form of mycoremediation. The key factors affecting mycoremediation have been largely discussed up to this point. However, when designing field-scale mycoremediation processes, more elements need to be pointed out. A scale-up factor of 10^6^ order of magnitude (from kg to 1000 t) is often reached. Expanding the size of a treatment pile (equal to a solid biological reactor) encompasses an order of 10^3^, while interfaces between air, water, and oily phases expand proportionally to their surfaces. Distances, such as those related to air pathways, hyphal length, and thickness causing pile stacking, scale linearly. The compaction effect of the soil also plays a role, increasing as the system grows. However, certain values, such as hyphal diameter, pore size, and grain size, remain unchanged regardless of the external size. To ensure a successful scale-up, each of these factors needs to be considered independently.

There are only a few instances of biopiles using bacteria and/or for PAH in the literature. Table 4 shows a summary of results and conditions for experiments using mycoremediation in different ways for PH-contaminated soils, most of them at lab-scale, showing the few examples of pilot/large-scale application of mycoremediation for TPH. Beškoski [221] conducted a bioremediation experiment for heavy residual fuel-oil-polluted soil. They inoculated fungi and bacteria in a biopile (75 m × 20 m with a height of 0.4 m). Re-inoculation was performed periodically with biomasses of microbial consortia isolated from the soil and with nutrients (N, P, and K). Aeration was improved by systematic mixing. The biopile was protected from direct external influences by a polyethylene cover. After 150 d, there were 96%, 97%, and 83% reductions in the aliphatic, aromatic, and nitrogen–sulphur–oxygen (NSO) asphaltene fractions, respectively [222].

In order to evaluate the general feasibility of field-scale mycoremediation, a laboratory treatability and or mesocosm study is required (see examples for PH-contaminated soils in Table 4). Promising results are visible at just 22 days for soils [223] and even earlier for liquid experiments [224]. The augmentation with the fungal species of Ascomycota and Basidiomycota lead to high PH depletion within short incubation (maximum 150 days). In the experiments run by [29,36,153], biostimulation controls have yielded a high percentage of PH depletion without fungal inocula. In these cases, fungi such as *Pleurotus* and *Agaricus* [131] facilitated a higher depletion of the heaviest PH fractions [131]. In [36] they observed instead a process of adhesion and later consequent degradation, which was enabled by the fungal hyphae. Refs. [36,153] observed an enhancement of the bacterial community and at the same time a reduction in the fungal one. The nutrients brought by fertilization, but even better by organic residues, which are slow-release and have a long-lasting effect, and by SMS and bulking agents stimulated the microbial and inoculated strains. By contrast, the addition of nutrients such as nitrogen at high extent can have a negative effect on the microorganisms, due to excessive ammonia production [203], so the C:N ratio could change from 10 to 27, as mentioned above.

As matter of fact, the dogmatic C:N ratio dogma of 10 might be revised and adapted to the different type of soils and fungi to be inoculated. Indeed, in Table 4, there are a number of examples, some of which stick to the dogma, while others differ completely: for example Robichaud et al. (2019) [153] achieved a maximum of 73% of TPH depletion with fungal inocula (*P. ostreatus*) with a 100:9 C:N ratio, while Covino [36] achieved 80% of degradation with biostimulation and 86% with *Pleorotus* sp. with a C:N ratio of 25. A hypothesis to support this statement would be that white-rot fungi might have experienced an unbalanced nutrient level in favor of carbon constituting the lignin, and, given that the production of ligninolytic enzymes occurs during the lignin degradation, they were doing it in the presence of a very low and even limiting concentration of nitrogen. On the other hand, Ascomycota achieved depletions rates between 40 and 96% with a C:N ratio closer to 10, including in their ecological niche, the soil, where nutrients are more heterogeneous. The addition of organic matter to the system has a generally positive effect, since it is a slow-release fertilizer [29] and can stimulate the indigenous microbial community to degrade the aliphatic hydrocarbons while fungi help in the enhancement of hydrocarbon availability [112].

Thus, the take-home message here is that, depending on the fungal inoculums to be applied to the soil, a prior assessment of the conditions must be performed taking into account the fungal specimen ecological niche and the relative need of nutrients to produce the enzymes of interest and/or act on the hydrocarbons.

The addition of organic matter is reasonable to a certain extent (in terms of nutrients). Ensuring a good level of nutrients in the soil is a good proxy for microbial activities, given that hydrocarbons bond to the organic matter of the soil, and good microbial activity, mostly from bacterial and fungal communities, will see to recycling and turnover of the organic matter in the soil. Managing the soil system, in terms of physical/chemical and microbiological parameters and the fungal inocula, and integrating one with the other may be pivotal for the overall process. To this end, the process might be closely followed by monitoring and in silico technologies, such as modeling, and an in-depth study of the microbial community from a taxonomical and functional point of view.

**Table 4:** Summary of several soil parameters affecting the mycoremediation of aliphatic hydrocarbons and the degradation achieved.

Reference	Texture	TOC	%O.M.	pH	TPHs (mg/kg)	%Degradation	Treatment	Scale	C:N:P	Time (Days)	T (°C)	Moisture	Inoculum
[112]	Loamy clay	13 g/kg		7.6	16,114	39.9	Native fungi	1.2 kg	100:10:01	120			germinated spores
						24.14	Biostimulation						
						2.7	Control						
[36]	silty clay	1.48		7.96	10,200	3.6	Control		100:4:0.9	60	28		lignocellulosic material
					9995	80.2	Control + inocula	150 g					
					9768	86.8	*P. ostreatus*						
					10,150	81.3	*B. rhodina*						
					9890	88.3	P + B						
[29]	silty clay	1.48		7.96	10,200	aprox 0	Control		100:4:0.9	60	28	50% WHC	lignocellulosic material
					9995	80.5	Control + inocula	150 g					
						aprox 80	Native fungi *Pseudoallescheria* sp.						
[225]			0.73		1200	aprox 16%	Control	300 g	100:39:137	28	30	10%	Liquid mycelia
						Aprox 55%	*Acinetobacter baumanni*i						
							*Talaromyces* sp.						
						65.6	A + T						
[226]						48.65	Native fungi 2						Liquid mycelia
						43.95	Native fungi 3	50 mL					(flasks)
						52.71	*Talaromyces* sp.						
						72.57	*Talaromyces* sp. + *A. baumannii*						
[131]	clay	5.44		7.6	24,000	10	10% Sterile straw		100:11:83	90	22–25		SMS
						71.5	*A. bisporus*	1 kg					
						69.5	*P. ostreatus*						
						57.7	*G. lucidum*						
[153]	sandy		1.5	7.72	2200	48	control	150–180 L	100:9:1	94	sub-artic clime		
						69	Compost						
						71	compost + willow						
						68	compost + *P. ostreatus*						spawn
						73	CWF						
						51	Fertilization (100/9/1)						
[227]					10%	79.9	genus Geomyces	500 g	100/10/1	30			104 spores mL^−1^
[203]	sandy loam		2	7.2	10,000	43	*Fusarium neocosmosporiellum*	300 g	100/10/1	150		60% WHC	1 cm^2^ MSM medium
[154]	sandy		2.3	8.66	18,000	48	*A. bisporus*	1 kg	100/10	40	20	70% WHC	SMS
						12	*P. eryngii*						
						29	*P. ostreatus*						
						34	*L. edodes*						
[228]	clay			7.2	54,074	47.6	*Lambertella* sp.	3 Kg	100:10:01	60		60% WHC	1 g/100 mL BSM
[229]				5	1 g/L	71.2 and 82.5	*Aspergillus sydowii* BOBA1		0.1% *w*/*v* NP	21	25		MSM + 0.1% SE oil (*v*/*v*)
[223]		4		7.9	69,000	aprox 25	Control	1 m3	soil1	98			
		5		7.75		aprox 85	*P. pulmonarius*		100/10/1				SMS
		4		8.75	54,000	12	Control		soil2	28			
		5		7.5		64	*P. pulmonarius*						SMS
	sandy loamy	3		7.9–8.6	12,000	40	*P. pulmonarius*			22	ambient		SMS
[230]			12.2	7.7	60,600	90	Bioaugmentation with *Rhizopus* sp.	53 g	100:10:1	35	30		Liquid
[224]				7.5		90%	*Trematophoma* sp. UTMC 5003	10 mL		15	28		Liquid
[231]					1000/ 15,000	98/ 40	*Fusarium* sp. F092	20 mL		60	25		Liquid
[108]					1% *v*/*v*	100	*Penicillium*	100 mL		60	25		Malt extract agar

While mycoremediation holds considerable promise, several challenges must be addressed to unlock its full potential. Understanding the complex interactions between fungi and contaminants and optimizing environmental conditions remain critical research areas. Exploring the functionality and adaptability of fungi in different ecosystems will help create tailored mycoremediation solutions for diverse contamination scenarios. As examples of this, several European research projects have been financed with the objective of developing mycoremediation as an efficient and realistic approach, including LIFE MySoil. This project also covers practical issues such as scaling up production and participation in the circular economy.

As research in mycoremediation continues to expand, existing techniques are being honed. Furthermore, large-scale implementation of mycoremediation techniques requires an in-depth understanding of the economic feasibility and regulatory frameworks involved. More projects covering the various aspects of the technology are needed in order to achieve more efficient and secure results and to promote mycoremediation as part of the best available technologies (BATs). Cost-effectiveness, scalability, and social acceptance of mycoremediation strategies must be carefully assessed to ensure their widespread use in waste management practices.

## 4. Conclusions

Mycoremediation is a promising technology for the decontamination of aliphatic petroleum hydrocarbons in soil, offering a more sustainable and environmentally friendly solution compared with other remediation technologies. However, this review shows that there are few experiences of mycoremediation at bench-scale, indicating a limited amount of research on this topic. There is still a lack of knowledge on the fungal degradation of aliphatic hydrocarbons, particularly in terms of the degradation pathways and the enzymes involved in the initial steps.

The factors that can determine the effectiveness of mycoremediation include the bioavailability of hydrocarbons; the selection of fungal species and bulking agents; the interaction between the inoculated fungi and the indigenous microbiota; soil properties and nutrients; and environmental factors such as humidity, oxygen, and temperature. These factors can be managed to improve the efficiency of mycoremediation through technological advances such as the structure of mycopiles, the application of surfactants, and the control of environmental factors.

Further research, innovation, and collaboration among scientists, policymakers, and industry stakeholders are crucial for realizing the full potential of mycoremediation in environmental restoration efforts.

## Figures and Tables

**Table 1:** Non-exhaustive extraction approaches for chemical methods used to estimate the bioavailable fraction of aliphatic hydrocarbons in soil.

**Remediation Approach**	**Extractant and Conditions**	**Notes**	**Reference**
Biostimulation of 7 aged soils (endpoint, 14 weeks)	*n*-propanol *n*-propanol: H_2_O (50:50) *n*-butanol. Orbital shaking (130 rpm) for 60′ with a solvent/soil ratio (10:1, *v*/*w*)	Relating residual C_12_-C_40_ concentrations following bioaccessibility assay with those after 14 weeks of biodegradation via linear regression models yielded best results with propanol: water (50:50) giving an R^2^ value of 0.96 and a slope of the best fit line equal to 1.08	[23]
Biostimulation of 7 aged soils (endpoint, 14 weeks)	40 mM β-HPCD in water. Orbital shaking (130 rpm) for 24 h with an extractant/soil ratio (20:1, *v*/*w*)	High correlation between residual C_12_-C_40_ concentrations following bioaccessibility assay and those after 14 weeks of biostimulation yielding an R^2^ value of 0.88 and a slope of the best fit line equal to 1.02.	[23]
Biostimulation of 9 aged soils (endpoint, 245 days)	40 mM β-HPCD in water. Orbital shaking (130 rpm) for 24 h at 20 °C with an extractant/soil ratio (20:1, *v*/*w*)	Residual C_10_-C_40_ hydrocarbons after the bioaccessibility assay were highly correlated with those obtained after 245 days of biostimulation yielding an R^2^ value of 0.94 and a slope of the best fit line equal to 1.02	[25]
Pilot-scale enhanced natural attenuation (endpoint, 320 days)	Same as [23] except for β-HPCD concentration (50 mM)	Linear regression models combining bioaccessibility vs. biodegradation enabled the prediction of degraded aliphatic hydrocarbons in pilot-scale plant	[24]
Soil slurry (endpoint, 84 days)	Soil extracted with 0.01 M CaCl_2_ solution (20:1, *v*/*w*) containing Tenax at a soil/sorbent 1.5:1 (*w*/*w*) ratio	TENAX extraction turned out to be a valuable method for the prediction of residual concentrations of total aliphatic hydrocarbons after biodegradation although a slight underestimation of the degradation of readily bioavailable hydrocarbons was observed	[27]
Biostimulation of 9 aged soils (endpoint, 245 days)	Soil extracted with 0.01 M CaCl_2_ solution (20:1, *v*/*w*) containing Tenax at a 1:1.2 (*w*/*w*) ratio	Residual C_10_-C_40_ hydrocarbons after the bioaccessibility assay were highly correlated with those obtained after 245 days of biostimulation yielding an R^2^ value of 0.92 and a slope of the best fit line equal to 0.77	[25]
Mycoaugmentation of an aged soil with indigenous strains (endpoint, 60 days)	Soil extracted by supercritical CO_2_ at 50 °C and 200 bar and desorbed hydrocarbons collected after different time intervals	Desorption data of total aliphatic hydrocarbons firmly fit the two-site model proposed by Williamson et al. (1998) [28]. The bioavailable threshold was trespassed in microcosms inoculated with *Pseudoallecheria* sp. for C_10_-C_14_ and C_21_-C_27_ fractions	[29]

**Table 3:** Bulking agent and inoculum media used for mycoremediation of PH-polluted soil.

Material	Soil: Bulking Agent Ratio (w:w)	Fungi	Reference
wheat straw: poplar wood chip (70:30 w:w)	1:0.2	*Pseudoallescheria* sp.	[29]
wheat straw: poplar wood chip (70:30 w:w)	1:0.2	*P. ostratus* and *B. rhodina*	[36]
Spent mushroom substrate	1:0.1	*A. bisporus*, *P. ostreatus*, and *G. lucidum*	[131]
Inoculum: 5% pure sawdust spawn of *P. ostreatus*, 7.1% sundried *Populus* spp. woodchips, and 2.9% naturalized *P. ostreatus* mycelium (*v*/*v*) Bulking agent: mature municipal compost	1:0.15 inoculum 1:0.15 compost	*P. ostreatus*	[153]
Spent mushroom substrate	1:0.10	*A. bisporus*, *P. ostreatus*, *P. eryngii*, and *L. edodes*	[154]
Spent mushroom substrate	1:0.075	*P. ostreatus*	[155]

## Data Availability

No new data were created or analyzed in this study. Data sharing is not applicable to this article.

## Supplementary Materials

The following supporting information can be downloaded at: https://www.mdpi.com/article/10.3390/jof9121205/s1, Figure S1: Cluster graph obtained through bibliometric mapping using VOSviewer and Web of Science database for studies obtained with the search words: “Mycoremediation” and “Aliphatic”; showing its network correlation.; Table S1: IP bibliography—Espacenet, using the search criteria mentioned in column 1.

## Abbreviations

Aliphatic Petroleum Hydrocarbons	(APH)
Petroleum Hydrocarbons	(PH)
Total Petroleum Hydrocarbons	(TPH)
Long Chain Hydrocarbons	(LCH)
Thermal Desorption	(TD)
Polycyclic Aromatic Hydrocarbons	(PAH)
Cytochrome P450	(P450 or CYP)
White Rot Fungus	(WRF)
Technology Readiness Levels	(TRL)
Carbon/Water Partition Coefficient	(K_oc_)
Hydrocarbons	C_10_-C_40_ (C_10_-_40_)
Unspecific Peroxygenases	(UPOs)
Organisation for Economic Cooperation and Development	(OECD)
Critical Micelle Concentrations	(CMC)
Dichloromethane	(DMC)
Spent Mushroom Substrate	(SMS)
Black Yeast-Like Fungi	(BYLF)
Free Air Space	(FAS)
Bacteria Fungi Interactions	(BFI)
Carbon:Nitrogen:Phosphorus Ratio	(C:N:P)
Next Generation Sequencing	(NGS)
Nonaqueous Phase Liquid	(NAPL)
Colony Forming Unit	(CFU)
Soil Organic Carbon	(SOC)
Internal Transcribed Spacer	(ITS)
Potato Dextrose Broth	(PDB)
