# Supplementary material for: Main Factors Determining the Scale-Up Effectiveness of Mycoremediation for the Decontamination of Aliphatic Hydrocarbons in Soil
Source: J Fungi (Basel). 2023 Dec 16;9(12):1205. doi: 10.3390/jof9121205 (PMC10745009; doi:10.3390/jof9121205)
Supplement: Supplementary file 1 [file jof-09-01205-s001.zip › jof-2746521-supplementary.pdf]

Supplementary material

Main Factors Determining the Scale-Up Effectiveness of Mycoremediation for the Decontamination of Aliphatic Hydrocarbons in Soil

Rafael Antón-Herrero †1, Ilaria Chicca †2, Carlos García-Delgado *3, Silvia Crognale 4, Davide Lelli 4, Romina Gargarello 5, Jofre Herrero 5, Anko Fischer 6, Laurent Thannberger 7, Enrique Eymar 1, Maurizio Petruccioli 4 and Alessandro D'Annibale 4.

1 Department of Agricultural Chemistry and Food Science, Universidad Autónoma de Madrid, Madrid, Spain
2 Novobiom, Ottignies-Louvain-la-Neuve, Belgium
3 Department of Geology and Geochemistry, Universidad Autónoma de Madrid, Madrid, Spain
4 Department for Innovation in Biological, Agri-food and Forestry Systems, University of Tuscia, Tuscia, Italy
5 Water, Air and Soil Unit, Eurecat - Technological Centre of Catalonia, Manresa, Spain
6 Isodetect, Leipzig, Germany
7 VALGO, Petit-Couronne, France
† These authors share the first authorship.
* Correspondence author: Carlos Garcia-Delgado: carlos.garciadelgado@uam.es

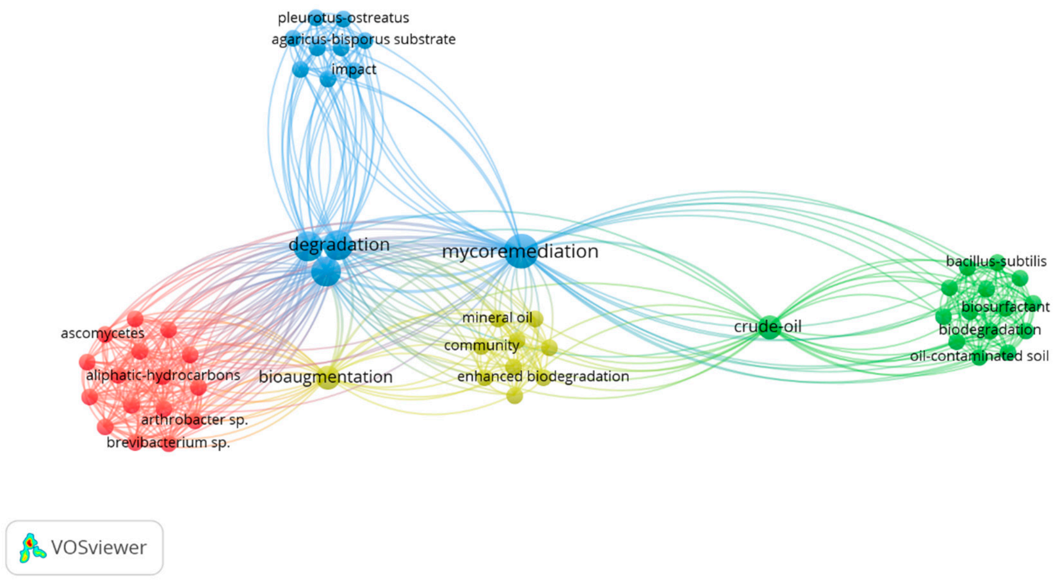

Figure S1. Cluster graph obtained through bibliometric mapping using VOSviewer and Web of Science database for studies obtained with the search words: "Mycoremediation" and "Aliphatic"; showing its network correlation.

Table S1. IP bibliography – Espacenet, using the search criteria mentioned in column 1.

Entry	Number	Title	Description	Authors	Year
**Mycoremediation**	US2008264858 (A1)	Delivery systems for mycotechnologies, mycofiltration and mycoremediation	Related upon the way of delivery, or spreading, several strains for numerous applications. Main claims concern the bag/cloth, as a tool. The activity of the strains is not the main topic.	STAMETS PAUL EDWARD [US]	2008
	US8759057 (B1)	Methods for purifying enzymes for mycoremediation	A process for purifying laccase from an ectomycorrhizal fruiting body is disclosed. The process includes steps of homogenization, sonication, centrifugation, filtration, affinity chromatography, ion exchange chromatography, and gel filtration.	CULLINGS KENNETH W [US]; DESIMONE JULIA C [US]; PAAVOLA CHAD D [US]	2014
	US2023014538 (A1)	*In-situ* mycoremediation system and process	This patent also concerns a device for the spreading of fungal inoculum, through a screened rod. It does not mention specific hydrocarbons treated.	GREGG JOHN [US]; GREGG LAURA [US]; GREGG JAMES [US]	2023
**Mycodegradation**	No Results				
**Fungal & remediation**	1 result regarding remediation of mycotoxin.				
**Fungi & remediation**	CZ2012156 (A3)	Remediation and reclamation process of contaminated areas using microorganisms and mycorrhizal fungi	Remediation process of soil contaminated with organic pollutants of crude oil and coke-chemical origin, by using cultures of microorganisms utilizing these pollutants with simultaneous or subsequent reclamation implemented by planting trees in connection with application of mycorrhizal fungi and seeding leguminous plants inoculated with rhizopheric bacteria that fix atmospheric nitrogen on roots of these plants.	MAREK MIROSLAV [CZ]; HORSAKOVA IVETA [CZ]; VRBA PETER [SK]; MAREK ALES [CZ]	2013
**Fungi & crude & oil**	CN105013813 (A)	Method for biologically remediating crude	The method comprises the following specific steps: fungi sticks are	LIAO XIANGRU; ZHANG YONG; TIAN QIAOPENG;	2015

		oil contaminated soil by using edible fungi residues	crushed to obtain fungi residues; enzyme production inducing liquid is added for cultivating under proper conditions to obtain a fermentation ripening system; then, the fermentation ripening system is added to the crude oil contaminated soil in a ratio of 1: 1-3: 1; and dual functions of microbial remediation and phytoremediation can be realized through the degradation of ligninase in a fungi residue fermentation system to phenolic compounds and organic fertilizers provided by the system. This is more related to enzyme and probiotic production.	CAI YUJIE; GUAN ZHENGBING	
**Fungi & hydrocarbon**	CN110153177 (A)	Method for treating PAH polluted soil by fungi	Strain Not specified, pollutant PAH.	LI QIQIAN; LUO CHUNLING; LI JUN; ZHANG GAN	2019
	CN109465293 (A)	Method for restoring petroleum hydrocarbon contaminated soil by using white rot fungi (WRF).	Petroleum contamination.	DAI BAIPING; ZHANG XIAOLIN; YAO WENCHONG; LIU KUN; WANG YOUJING; PAN YANSHUO; KUANG HONGQIANG	2019
	CN106745800 (A)	Method for biologically degrading n phenanthrene by using *Aspergillus niger* fungi.	Strain of *A. nige*r and for tH phenanthrene	WANG RUNAN	2017
	CN101935683 (A)	Method for measuring PAH degrading capability of WRF.	WRF Strain and for Petroleum HC.	XUANZHEN LI XIANGUI LIN	2010
	CN113755337 (A)	Fungi GIG-1 and GIG-2 for degrading PAH in petroleum-contaminated soil and mixed bacterial agent	*Cephalotrichum dendrocephalum +Scedosporium dehoogii* strains for PAH.	LI JIBING; LUO CHUNLING; DAI YELIANG; ZHAO XUAN -	2021

		and application thereof.			
**Fungi & aliphatic**	7 results: none for remediation of aliphatic hydrocarbons, only production or microbial inhibition/control.				
**Fungi & availability**	2 results for rumen species *Neocallimastix*.				
**Crude & oil & availability**	No Results				
**Hydrocarbon & availability**	DE4238430 (A1)	Microbial clean-up of hydrocarbon-contaminated soil - by adding complexing agent, pref. cyclodextrin, to increase bio-availability of hydrocarbons	Focused on PAH and microorganisms are not defined.	MUELLER-MARKGRAF WOLFGANG DR [DE]; GLASER ANDREAS [DE]; BRONNENMEIER REINHOLD DR [DE]	1994
